# KAS-CUT&Tag for direct mapping of transcription bubbles

**DOI:** 10.64898/2026.05.15.725569

**Published:** 2026-05-19

**Authors:** Weifang Wu, Jacob E. Greene, Kami Ahmad, Steven Henikoff

**Affiliations:** 1 Basic Sciences Division, Fred Hutchinson Cancer Center, Seattle, WA, USA.; 2 Molecular Medicine and Mechanisms of Disease PhD Program, University of Washington, Seattle, WA, USA.; 3 Howard Hughes Medical Institute, Chevy Chase, MD, USA.

## Abstract

Transcription by RNA Polymerase II (Pol II) generates dynamic transcription bubbles as it moves forward, but existing methods map transcriptional activity *in vivo* only indirectly. We introduce KAS-CUT&Tag, a method that combines N_3_-kethoxal labeling of exposed guanines with CUT&Tag to directly map transcription bubbles. We find that bubble density varies across Pol II-bound genes at transcription start sites, gene bodies, and termination sites. Transcription bubbles are enriched at genes marked by the H3K36me3 histone modification and chromatin-bound splicing factor U2AF2, but the highest enrichment is at replication-coupled histone genes throughout the cell cycle. KAS-CUT&Tag also detects colocalization of the histone gene transcription factor NPAT with Pol II at the Histone Locus Body, suggesting NPAT is juxtaposed to transcription bubbles via interaction with Pol II. Together, KAS-CUT&Tag enables direct mapping of Pol II and regulatory interactions at transcription bubbles, providing a powerful tool for precise analysis of transcriptional activity.

## Introduction

RNA polymerase II (Pol II) transcribes all protein-coding genes and many non-coding RNAs in the eukaryotic nucleus. Transcription begins with recruitment of unphosphorylated Pol II to promoters, forming a closed Pre-Initiation Complex (PIC) with general transcription factors. Transcription initiates with DNA unwinding at the promoter to form an open PIC with a single-stranded transcription bubble, facilitated by TFIIH and other general transcription factors^1–4^. Following transcriptional initiation, Pol II is phosphorylated on serine-5 (Pol II-Ser5p) of the largest subunit RPB1 C-Terminal Domain (CTD) heptapeptide repeats^5–7^ and then moves forward, but often pauses ~20–60 nucleotides downstream of the transcription start site (TSS)^8,9^. Release of this paused complex into active elongation is regulated by CDK9-mediated phosphorylation of the Pol II CTD at serine-2 (Pol II-Ser2p)^10,11^. After Pol II proceeds through the body of genes, transcription is terminated and Pol II disengages from its DNA template, whereupon the transcription bubble reanneals^12,13^.

Pol II translocates the single-stranded transcription bubble as it moves forward. Within this transcription bubble, the nascent RNA pairs with the template DNA as an RNA–DNA hybrid, positioning the RNA 3′ end in the Pol II catalytic site, while the non-template single-stranded DNA (ssDNA) is displaced^14,15^. As Pol II translocates, it exposes the next template base for pairing with an incoming nucleoside triphosphate, extending the RNA by one nucleotide^16^. Thus, the transcription bubble moves dynamically as Pol II progresses.

To map these ssDNA structures, we developed KAS-CUT&Tag, a method that combines N_3_-kethoxal labeling of exposed guanines in single-stranded bubbles with CUT&Tag. Applied to Pol II, KAS-CUT&Tag maps transcription bubbles at Pol II-bound sequences across the genome and shows that bubble density varies within and between loci. Perturbation experiments confirm that these bubbles arise from actively engaged Pol II. Pol II is highly enriched at replication-coupled histone genes and precisely co-localizes with the transcription factor NPAT. We conclude that KAS-CUT&Tag enables high-resolution mapping of transcription bubbles and anticipate broad applications in dissecting transcriptional regulatory mechanisms.

## Results

### KAS-CUT&Tag detects single-stranded DNA bound by Pol II

**Fig.1:**
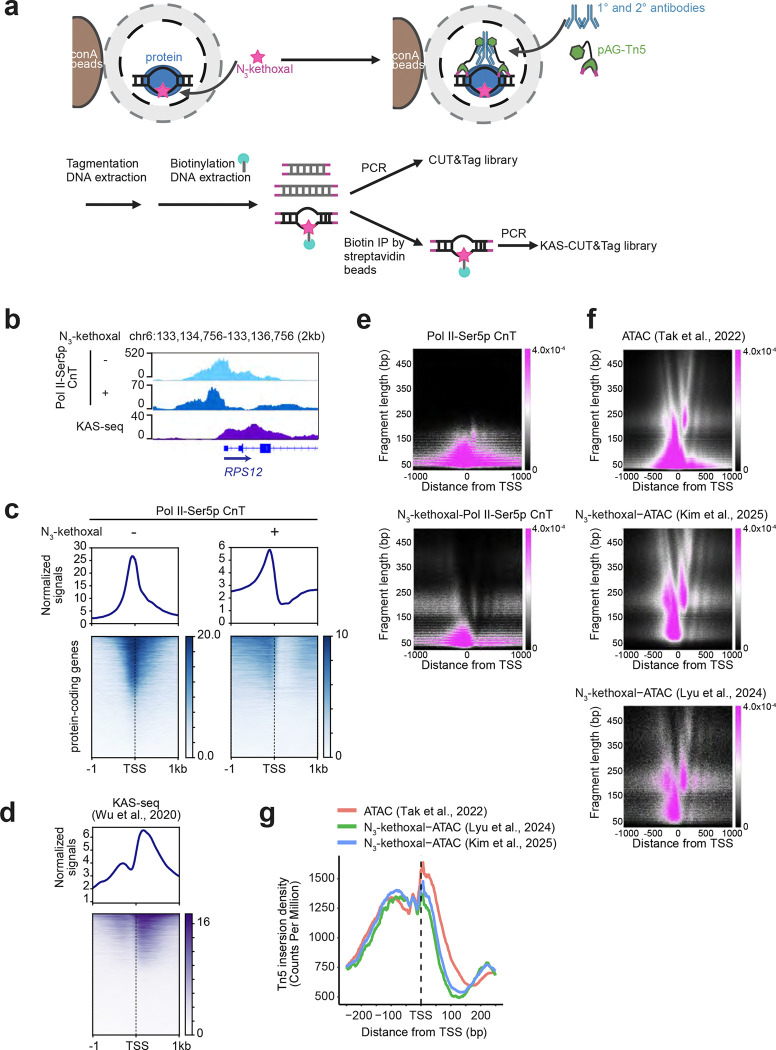
KAS-CUT&Tag detects single-stranded DNA bound by Pol II. **a,** Schematic of the KAS-CUT&Tag workflow (created with BioRender.com). N_3_-kethoxal (magenta star) labels ssDNA, while a primary antibody (blue Y-shape) binds the target protein (blue oval). A secondary antibody (blue Y-shape) enhances recruitment of the pAG-Tn5 transposome (green), which is activated by Mg^2+^ to insert adapters (magenta lines) at protein-bound sites. After DNA purification, adapter-ligated fragments are biotinylated via click chemistry. Biotinylated DNA is either amplified by PCR to generate the input CUT&Tag library, or immunoprecipitated with streptavidin beads before PCR to construct the KAS-CUT&Tag library. **b,** Coverage-normalized Pol II-Ser5P CUT&Tag (CnT) signals (±N_3_-kethoxal), shown alongside KAS-seq data from Wu et al in K562 cells^17^. Input CUT&Tag library corresponds to N_3_-kethoxal-treated CUT&Tag (CnT). **c, d,** Heatmaps (bottom) and average plots (top) centered on the TSSs of 12,397 protein-coding genes, showing Pol II-Ser5P CnT (±N_3_-kethoxal) (**c**) and KAS-seq data in K562 (**d**). Each row represents one gene. **e, f,** V-plots showing fragment size versus distance from TSSs for Pol II-Ser5P CnT (±N_3_-kethoxal) (**e**) and ATAC-seq (±N_3_-kethoxal) (**f**). ATAC-seq (−N_3_-kethoxal) is from Tak et al^18^, while ATAC-seq (+N_3_-kethoxal) is from Kim et al^19^ and Lyu et al^65^. **g,** Density plot of Tn5 fragment ends within a 400 bp window centered on TSSs.

To efficiently map single-stranded transcription bubbles, we capitalized on N_3_-kethoxal-assisted single-stranded DNA sequencing (KAS-seq), where reaction of unpaired guanine nucleosides with N_3_-kethoxal is followed by biotin labeling and immunoprecipitation^17^. Cells are immobilized on Concanavalin A (ConA) beads and permeabilized, followed by a 10-minute treatment with N_3_-kethoxal. We then proceeded with antibody-tethered Tn5 tagmentation, fragment release, and DNA purification. This DNA is then biotinylated via click chemistry, and biotinylated DNA is immunoprecipitated with streptavidin beads and subjected to PCR for library construction. Parallel reactions omitting the immunoprecipitation step serve as input controls. We call this general method kethoxal-assisted single-stranded DNA assay for CUT&Tag (KAS-CUT&Tag) (Fig. 1a).

We first applied KAS-CUT&Tag using antibodies against Pol II and Ser2-phosphorylated Pol II in human K562 cells. We optimized the immunoprecipitation step, yielding highly specific and reproducible KAS-CUT&Tag profiles (Fig. S1a–d). KAS-CUT&Tag profiles correlated with input controls over gene bodies but not with GC content (Fig. S1e,f), confirming that the labeling method was not biased. We found that light crosslinking (0.1% formaldehyde for 1 min) after N_3_-kethoxal treatment, followed by tagmentation under low-salt CUTAC conditions, increased KAS-CUT&Tag library yields by ~9-fold while maintaining high reproducibility (Fig. S2a–c).

We next examined whether N_3_-kethoxal treatment affects CUT&Tag profiles by analyzing KAS-CUT&Tag input libraries, which represent N_3_-kethoxal-treated CUT&Tag. Compared to untreated CUT&Tag, N_3_-kethoxal-treated CUT&Tag for Pol II-Ser5P showed signal depletion from the TSS to ~+500 bp (Fig. 1b,c). Within this gap, KAS-seq ssDNA signals were enriched (Fig. 1b,d), thus N_3_-kethoxal labeling of unpaired guanines in ssDNA appears to prevent reannealing, thereby blocking Tn5 tagmentation in this region and producing the observed loss of signal. V-plots supported this interpretation: Untreated Pol II-Ser5P CUT&Tag showed dense subnucleosomal fragments around the TSS, whereas N_3_-kethoxal-treated CUT&Tag displayed a pronounced loss of subnucleosomal fragments and only a modest increase in mononucleosomal fragments downstream, consistent with the observed gap (Fig. 1e). To test whether blocking of Tn5 tagmentation by N_3_-kethoxal is a general limitation, we compared untreated ATAC-seq^18^ and N_3_-kethoxal-treated ATAC-seq (KAS-ATAC input) from published data^19,20^. In contrast to CUT&Tag, where Tn5 integration is tethered to antibody-bound chromatin, ATAC-seq utilizes freely diffusing Tn5 that targets accessible double-stranded DNA (dsDNA). V-plots of N_3_-kethoxal-treated ATAC-seq data from two different laboratories^19,20^ revealed a loss of subnucleosomal fragments around the TSS (Fig. 1f). In addition, insertion-site analysis showed marked depletion downstream of the TSS (Fig. 1g), consistent with Tn5 exclusion from ssDNA in this region. Together, these results indicate that N_3_-kethoxal blocks Tn5 integration into ssDNA between the TSS and the +1 nucleosome, leading to a loss of short fragments while preserving longer fragments derived from adjacent dsDNA (Fig. S3a). These results demonstrate that KAS-CUT&Tag enables specific and sensitive profiling of single-stranded DNA generated by Pol II.

### KAS-CUT&Tag maps transcription bubbles

**Fig.2:**
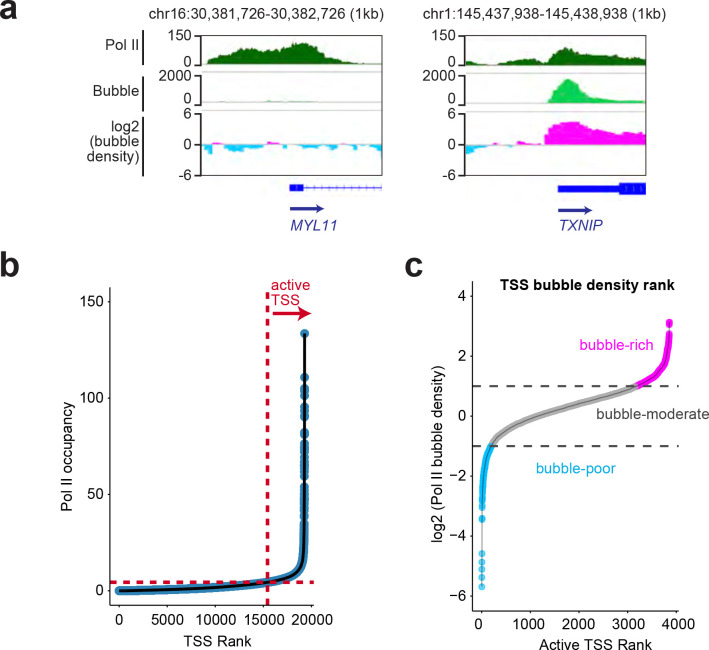
Transcription bubble density varies between gene promoters. **a,** Coverage-normalized signals for Pol II (CnT), transcription bubbles (KAS-CnT), and log_2_(bubble density), calculated as log_2_(Pol II KAS-CnT / CnT), in K562 cells. **b,** Ranking of 19,294 protein-coding gene TSSs by Pol II occupancy in K562, quantified by Pol II CnT signal in K562. **c,** Ranking of 3,859 active gene TSSs by bubble density, quantified by log_2_(Pol II KAS-CnT/CnT) ratios in K562 cells.

Pol II-targeted KAS-CUT&Tag input profiles chromatin-bound Pol II, while Pol II-targeted KAS-CUT&Tag IP captures transcription bubbles. We used the ratio of KAS-CUT&Tag to CUT&Tag signal as a measure of transcription bubble density, with higher values indicating a greater fraction of Pol II localized in transcriptional bubbles. This ratio is normalized to CUT&Tag signal, thus it also controls for Tn5 insertion artifacts such as loss of subnucleomal fragments around TSSs (Fig. 1e). Surprisingly, we found that bubble density varied substantially around gene TSSs bound by Pol II. For example, although Pol II occupancy was similar around the TSSs of *MYL11* and *TXNIP*, bubble density was markedly higher at *TXNIP* (Fig. 2a). This implies that at genes such as *MYL11*, only a fraction of chromatin-bound Pol II is in an open complex.

To systematically identify genes with high or low bubble density around their TSSs, we analyzed 3,859 active genes with high Pol II occupancy within a 2-kb window centered on their TSSs (Fig. 2b). Among these genes, 17% (674 TSSs) are bubble-rich (log_2_ bubble density > 1), while 5% (194 TSSs) are bubble-poor (log_2_ bubble density < −1) in K562 cells (Fig. 2c). For instance, bubble-rich *SMIM30*, bubble-moderate *GGPS1* and bubble-poor *LY6G6F* TSS all showed similar Pol II occupancy levels (Fig. S4a). This indicates that the proportion of promoter-bound Pol II localized in an open complex varies between active genes.

**Fig.3:**
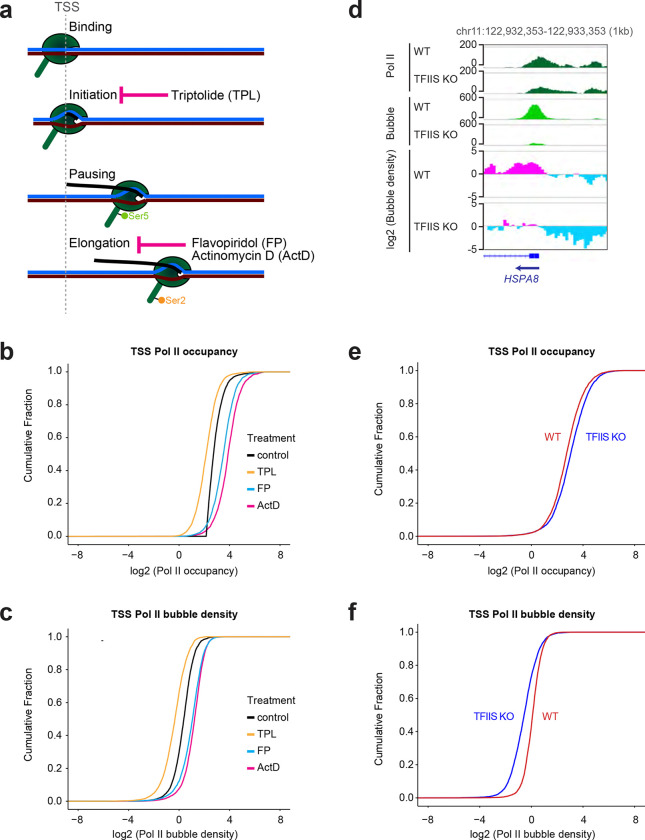
KAS-CUT&Tag maps transcription bubbles generated by Pol II. **a,** Diagram illustrating stages of the transcription cycle targeted by different transcription inhibitors. **b,** Cumulative plots showing Pol II occupancy at TSSs in K562 cells under the indicated treatments, quantified by Pol II CnT signals. **c,** Cumulative plots showing Pol II bubble density at TSSs under the same conditions, quantified by log_2_(Pol II KAS-CnT/CnT) ratios. **d,** Coverage-normalized signals for Pol II (CnT), bubbles (KAS-CnT), and log_2_(bubble density), calculated as log_2_(Pol II KAS-CnT / CnT), in WT and TFIIS KO HEK293T cells. **e,** Cumulative plots showing Pol II occupancy at TSSs in WT and TFIIS KO cells. **f,** Cumulative plots showing Pol II bubble density at TSSs in WT and TFIIS KO cells.

We hypothesized that variation in promoter-proximal Pol II pausing contributes to differences in bubble density, with paused Pol II exposing single-stranded DNA that is reactive to N_3_-kethoxal. To test this, we treated K562 cells with transcription inhibitors that target distinct stages of the transcription cycle (Fig. 3a). Triptolide (TPL) inhibits transcription initiation by targeting the XPB subunit of TFIIH, which catalyzes promoter opening^21^. Flavopiridol (FP) blocks pause release by inhibiting cyclin-dependent kinases^22^, including CDK9, which phosphorylates Pol II serine-2. Actinomycin D (ActD) intercalates into DNA and stalls Pol II elongation^23^. We validated drug action by profiling Pol II occupancy using CUT&Tag. As expected, TPL treatment led to a global reduction in Pol II occupancy due to impaired loading (Fig. 3b; Fig. S4b), which was accompanied by a corresponding decrease in bubble density (Fig. 3c; Fig. S4c). In contrast, FP and ActD treatments increased Pol II occupancy at TSSs due to blocked pause release (Fig. 3b; Fig. S4b), and both also elevated bubble density at TSSs (Fig. 3c; Fig. S4c). These results confirm that KAS-CUT&Tag detects transcription bubbles caused by Pol II activity.

To better understand how KAS-CUT&Tag detects transcriptionally active Pol II, we examined Pol II backtracking, a state in which the RNA 3′ end is displaced from the catalytic site^24–26^. The elongation factor TFIIS reactivates backtracked Pol II by promoting cleavage of the extruded RNA, allowing realignment of the 3′ end^24–26^. We profiled wild-type (WT) and TFIIS-knockout (KO) HEK293T cells. If transcription bubbles arise from active Pol II, loss of TFIIS should trap Pol II in an inactive state and reduce bubble density without altering total occupancy. Pol II is known to backtrack at heat shock genes and requires TFIIS for reactivation^27–29^. Accordingly, the promoter of the *HSPA8* heat shock gene showed high bubble density in WT cells, but this signal was reduced in TFIIS KO cells despite only moderate change in Pol II occupancy (Fig. 3d). Genome-wide analysis confirmed that bubble density at TSSs broadly declines despite slightly increased Pol II occupancy (Fig.3e,f). These findings demonstrate that KAS-CUT&Tag enables mapping of transcription bubbles and that TFIIS maintains these bubbles.

### Transcription bubble density varies across gene bodies and termination sites

Pol II escapes promoter pausing upon serine-2 phosphorylation of its CTD repeats and subsequently elongates across gene bodies^10,11^. We quantified Pol II-Ser2p occupancy and transcription bubbles using coverage-normalized Pol II-Ser2p CUT&Tag and KAS-CUT&Tag signals, respectively, and calculated bubble density as the log_2_ ratio of Pol IIS2P KAS-CUT&Tag to CUT&Tag across gene bodies. In contrast to the incompatibility between N_3_-kethoxal treatment and Tn5 tagmentation around TSSs, we observed minimal impact of N_3_-kethoxal on CUT&Tag profiles of elongating Pol II-Ser2P, which remained largely unchanged across gene bodies (Fig. S3b,c).

**Fig.4:**
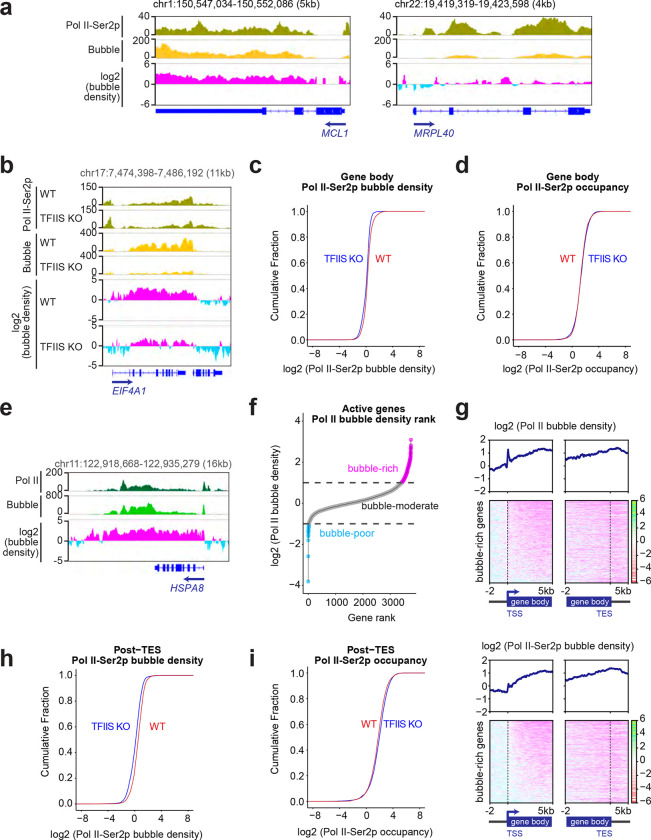
Bubble density varies across gene bodies and termination sites. **a,** Coverage-normalized signals for Pol II-Ser2p CnT, KAS-CnT, and log_2_(Pol II-Ser2p KAS-CnT/CnT) ratios in K562 cells. **b,** Coverage-normalized signals for Pol II-Ser2p CnT, KAS-CnT, and log_2_(Pol II-Ser2p KAS-CnT/CnT) in WT and TFIIS KO cells. **c, d,** Cumulative plots showing Pol II-Ser2P bubble density (c) or occupancy (d) across gene bodies in WT and TFIIS KO cells. **e,** Coverage-normalized signals for Pol II CnT, KAS-CnT, and log_2_(Pol II KAS-CnT/CnT) ratios in K562 cells. **f,** Ranking of 3,754 active genes by Pol II bubble density across entire transcription units (TSS to 2 kb downstream of TES), quantified by log_2_(Pol II KAS-CnT/CnT) ratios. **g,** Heatmaps (bottom) and average plots (top) aligned to the TSSs and TESs of 343 active genes in K562, showing log_2_(Pol II bubble density) or log_2_(Pol II-Ser2p bubble density). Each row represents one gene. **h,i,** Cumulative plots showing Pol II-Ser2P bubble density (h) or occupancy (i) across post-TES regions in WT and TFIIS KO cells.

Interestingly, as we observed at TSSs, transcription bubble densities varied substantially between active genes. For example, although the *MCL1* and *MRPL40* genes had similar Pol II-Ser2p occupancy across their gene bodies, *MCL1* had markedly higher bubble density (Fig. 4a). To systematically assess these gene body patterns while avoiding promoter-proximal signals, we quantified Pol II-Ser2p bubble density across 3,754 active genes ≥1.5 kb in length, considering the gene body as the region from +1 kb downstream of the TSS to the transcription end site (TES). Only 9% (342) of gene bodies were classified as bubble-rich, compared to 17% of active TSSs in K562 cells (Fig. S5a; Fig. 2c). As expected, inhibiting CDK9-dependent pause release with flavopiridol reduced both Pol II-Ser2p occupancy and bubble density in gene bodies (Fig. S5b,c). We found that Pol II-Ser2p occupancy in TFIIS KO cells remained similar to WT cells at the *EIF4A1* gene, but bubble density was reduced (Fig. 4b). However, genome-wide analysis showed that both Pol II-Ser2p occupancy and bubble density were similar between WT and TFIIS KO cells (Fig. 4c,d). These results suggest that gene-body Pol II-Ser2p includes both active and inactive elongating polymerase, and that KAS-CUT&Tag distinguishes the actively engaged fraction.

We assessed whether bubble density remains uniform across full transcription units. Pol II bubble density across the *HSPA8* heat shock gene was consistent from TSS to transcription end sites (TESs) in wild-type HEK293T cells (Fig. 4e). To assess this genome-wide, we used Pol II-targeted profiles to measure chromatin-bound Pol II, and calculated bubble density across entire transcription units, from TSS to 2 kb downstream of the TES. We found that 9% (343 of 3,754) of active genes were bubble-rich throughout the entire transcription unit (Fig. 4f). At these genes, Pol II and Pol II-Ser2p bubble densities were elevated at the TSS and remained relatively stable through gene bodies and post-TES regions (Fig. 4g). Overall, these findings reveal a subset of genes with uniformly high bubble density across their entire transcribed regions.

Finally, we examined transcription beyond annotated TESs, where Pol II often continues elongation despite cleavage and polyadenylation. We defined the 2-kb region downstream of TESs as post-polyadenylation regions and found that 37% (1,382) were bubble-rich in K562 cells (Fig. S5d). In TFIIS KO cells, bubble density declined in these regions without changes in Pol II-Ser2p occupancy (Fig. 4h,i), indicating that Pol II remains at transcription bubble downstream of TESs in a TFIIS-dependent manner. These findings highlight widespread heterogeneity in bubble density at Pol II-Ser2p transcribed genes.

### Transcription bubbles are enriched at genes marked with the H3K36me3 histone modification and the U2AF2 splicing factor

**Fig.5:**
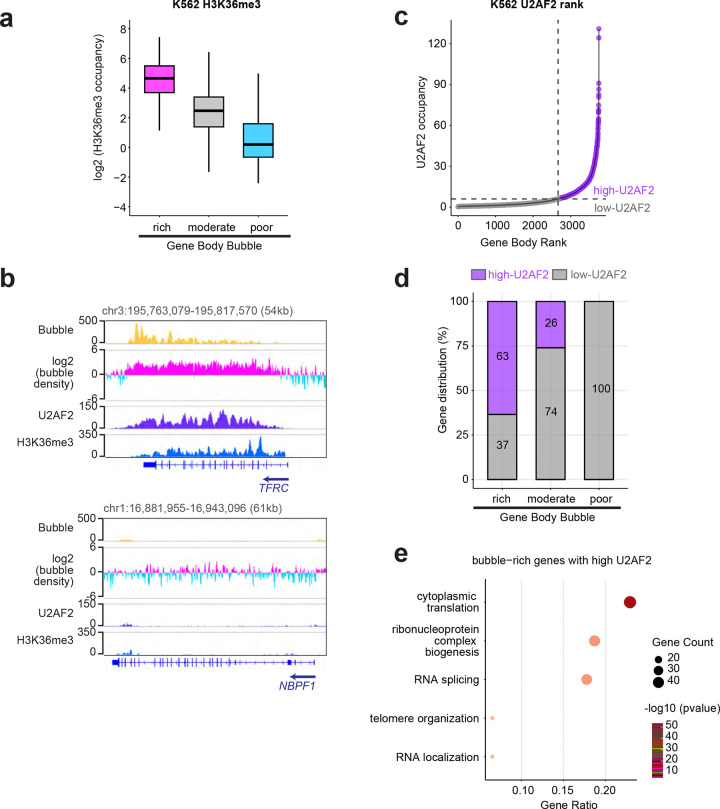
Transcription bubbles are enriched at gene bodies marked by the H3K36me3 mark and the U2AF2 splicing factor. **a,** Coverage-normalized H3K36me3 CnT signals across bubble-rich, -moderate, and -poor gene bodies in K562 cells. **b,** Coverage-normalized signals for Pol II-Ser2P KAS-CnT, log_2_(Pol II-Ser2p KAS-CnT/CnT) ratios, U2AF2 CUT&RUN (CnR) and H3K36me3 CnT in K562. **c,** Ranking of 3,754 active genes by U2AF2 CnR signal across gene bodies. **d,** Proportion of genes with high or low U2AF2 signal within bubble-rich, -moderate, and -poor groups. **e,** Dot plots showing the top five GO terms enriched in bubble-rich genes with high U2AF2 binding. Dot size represents gene count; color indicates adjusted −log_10_(p-value) using the Benjamini-Hochberg method.

Highly active gene bodies are often marked with histone H3 lysine 36 trimethylation (H3K36me3)^30,31^. To test if this modification might be associated with actively engaged Pol II, we compared the distributions of the H3K36me3 mark with bubble density generated by Pol II-Ser2p. Indeed, the H3K36me3 modification was elevated at bubble-rich gene bodies in both K562 and HEK293 cells (Fig. 5a; Fig. S6a).

The H3K36 methyltransferase SETD2^32^ binds elongating Pol II^33,34^, and deposits H3K36me3 to nucleosomes partially unwrapped by the Pol II transcription machinery^35^. One function of the H3K36me3 modification is to recruit the U2AF2 splicing factor that travels with elongating Pol II to chromatin^36^. This chromatin-bound U2AF2 preferentially binds highly expressed genes to enhance co-transcriptional splicing^36^. Comparing the distributions of Pol II-Ser2p bubble density, H3K36me3, and U2AF2 binding reveals that these three features co-occupy many genes in K562 cells. For example, the *TFRC* gene had high Pol II-Ser2p bubble density, H3K36me3, and U2AF2 binding throughout (Fig. 5b). In contrast, the *NBPF1* gene had little Pol II-Ser2p bubble density, H3K36me3, or U2AF2. Genome-wide analysis confirms that U2AF2 occupancy was high in bubble-rich gene bodies (Fig. S6b). We found that 63% (217 of 342) of bubble-rich and 26% (872 of 3,349) of bubble-moderate gene bodies showed high U2AF2 occupancy, compared to none of the bubble-poor gene bodies (Fig. 5c,d). Gene Ontology analysis on the 217 bubble-rich genes with high U2AF2 found that 18% of these genes are associated with RNA splicing (Fig. 5e).

These features also coincide in HEK293 cells. The expressed *HNRNPU* gene showed a high bubble density, H3K36me3 and U2AF2 occupancy, while the similarly active *ZDHHC16* gene lacks these features (Fig. S6c). Genome-wide, U2AF2 levels were ~10-fold higher at bubble-rich and 4-fold higher at bubble-moderate gene bodies than at bubble-poor genes (Fig. S6d). These findings suggest that Pol II-Ser2p generates more transcription bubbles at genes marked by H3K36me3 and bound by U2AF2.

### Transcription bubbles remain high at replication-coupled histone genes throughout interphase

**Fig.6:**
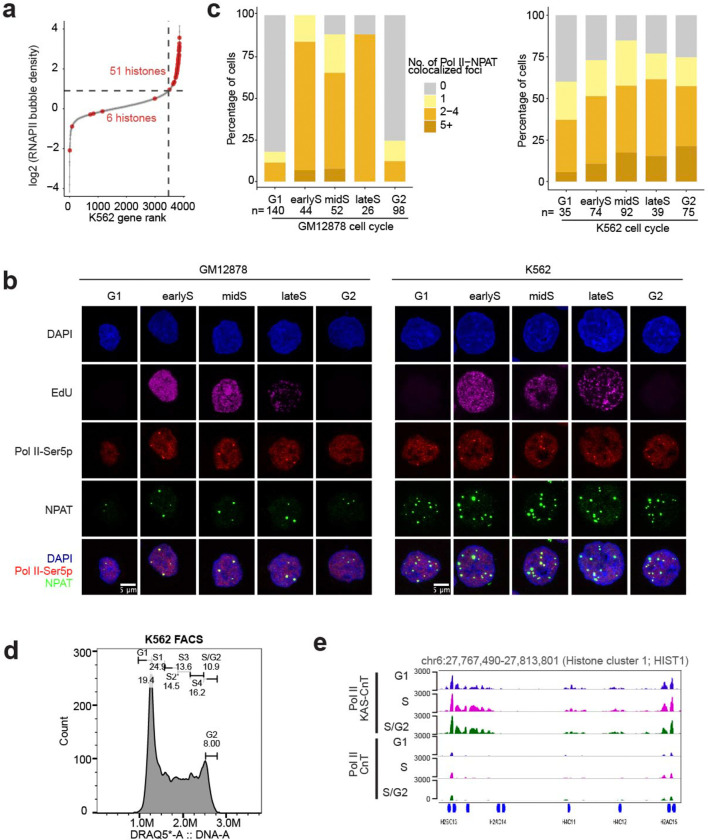
Transcription bubbles remain high at replication-coupled histone genes throughout interphase. **a,** Ranking of bubble density (log_2_(Pol II KAS-CnT/CnT) ratios) in 3,859 active genes from TSS to TES in K562 cells. **b,** Immunostaining of human GM12878 and K562 cells for the HLB factor NPAT (green) and Pol II-Ser5P (red), with EdU detected by click chemistry (magenta). For display, identical minimum and maximum intensity settings were applied to all images within each cell type and channel. At least 300 cells were analyzed. NPAT–Pol II-Ser5P colocalization foci were observed mainly in S-phase GM12878 cells, but throughout interphase in K562 cells. **c,** Distribution of GM12878 and K562 cells across the indicated cell-cycle stages by number of NPAT–Pol II-Ser5P colocalized foci. N indicates the number of cells analyzed at each stage. **d,** Isolation of cell cycle fractions. FACS gating schema to purify G1, S-phase, and G2 populations of K562 cells. Even-width gates were fit to the distribution of DNA content in exponentially proliferating K562 cells. Mitotic cells were gated out based on DRAQ5 eccentricity, as in Schraivogel et al.^66^ Percent of the parent population is shown for each gate. G1 cells were defined as left of the first mode. S-phase cells were collected from the gate labeled S3. S/G2 cells were collected from the last gate. Post-hoc analysis by fitting a gate right of the second mode (labeled G2 only) suggested that 8.0% of cells are in G2-phase, indicating 73% G2 purity in the S/G2 population. **e,** Coverage-normalized signals for Pol II KAS-CnT and CnT in G1, S, and S/G2-phase K562 cells.

To identify genes with high transcription bubble density, we ranked the 3,859 active genes by bubble density. Remarkably, 90% (51/57) of active replication-coupled histone genes ranked within the top 10%, indicating exceptionally high transcription bubble density (Fig. 6a). These genes are prone to Pol II backtracking and require TFIIS for proper gene expression^27^, and indeed we find that TFIIS is required to maintain transcription bubbles at these promoters (Fig. S7a).

Replication-coupled histone genes are assembled within Histone Locus Bodies (HLBs), nuclear compartments that require NPAT for their formation and function. To examine cell-cycle regulation of histone gene transcription, we immunostained K562 and GM12878 cells for NPAT and the initiating form of Pol II (Pol II-Ser5P), and used EdU labeling to identify S-phase cells. EdU staining patterns were used to further classify S-phase cells as early, mid, or late S phase (Fig. 6b). Among EdU-negative cells, those with NPAT foci numbers comparable to those in S-phase cells were classified as G2, and the remaining interphase cells as G1 (Fig. S7b–e). Approximately 33% of GM12878 cells were in S phase, compared with about 66% of K562 cells (Fig. S7f,g). In GM12878, approximately 90% of S-phase cells contained at least one colocalized Pol II-Ser5P and NPAT focus, whereas only about 20% of G1 and G2 cells showed colocalization (Fig. 6c), indicating that initiating Pol II accumulates at histone genes within HLBs mainly during S phase. In contrast, Pol II-Ser5P colocalized with NPAT in approximately 75% of S-phase K562 cells, 60% of G1 cells, and 75% of G2 cells (Fig. 6c), indicating that initiating Pol II remains associated with histone genes throughout interphase in K562 cells.

To assess whether Pol II remains localized to transcription bubbles at histone genes throughout interphase in K562 cells, we isolated G1, S, and S/G2 populations by FACS based on DNA content, and then performed Pol II CUT&Tag and KAS-CUT&Tag (Fig. 6d). Consistent with the imaging results, Pol II CUT&Tag signals were enriched at histone genes throughout interphase. In addition, at all stages, Pol II KAS-CUT&Tag signals at histone promoters were stronger than Pol II CUT&Tag signals (Fig. 6e), indicating that Pol II remains at transcription bubbles even when histone genes are not actively transcribed. Thus, formation of a transcription bubble alone is insufficient for gene expression. Instead, Pol II appears paused outside S-phase and is released into productive elongation specifically during S-phase, consistent with prior observations of short, aborted transcripts in G1/G2 and full-length histone mRNAs only in S-phase^37^.

### Transcription factor NPAT is juxtaposed to transcription bubbles at the Histone Locus Body

**Fig.7:**
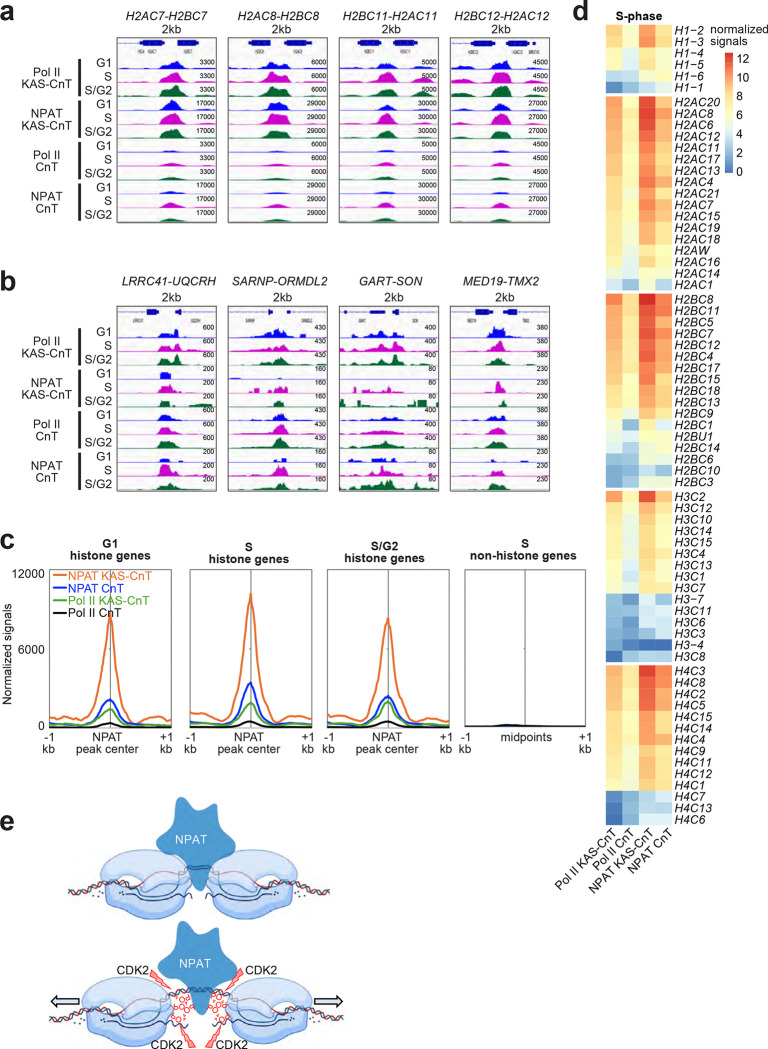
NPAT is juxtaposed to transcription bubbles at the Histone Locus Body. **a, b,** Coverage-normalized signals for Pol II and NPAT KAS-CnT and CnT at 4 representative bidirectional H2A/H2B gene pairs (b) and 4 bidirectional non-histone gene pairs (c) in K562 cells. **c,** Average plots centered on 36 NPAT KAS-CnT peaks at the 5′ ends of RD-histone genes, showing Pol II and NPAT CnT and KAS-CnT signals in G1, S, and S/G2 phase FACS-isolated K562 cells. An average plot centered on the midpoints of 12 bidirectional non-histone gene pairs in S phase cells is shown on the same scale to indicate background signals. **d,** Heat maps showing coverage-normalized Pol II and NPAT KAS-CnT and CnT signals at individual RD histone genes in S-phase K562 cells. Rows were sorted by Pol II KAS-CnT signal within each histone type. **e**, Model for the transcriptional activation of histone genes by protein phosphorylation. Two Pol II complexes are engaged with divergent histone gene promoters and bound to NPAT through IDR interactions that prevent Pol II progression. Upon cell cycle progression to the G1/S restriction point, activation of the CDK2 kinase phosphorylates both NPAT and Pol II CTDs and these negative charges disrupt IDR interactions, releasing Pol II for transcription.

NPAT is the transcription factor that specifically activates the Replication-coupled histone genes when phosphorylated by the CDK2 kinase at the G1/S transition^38^. Pol II precisely colocalizes with NPAT in K562 cells^39^. NPAT is a largely disordered 1427 aa protein^40^, and the Pol II CTD is also a disordered region. As intrinsically disordered regions (IDRs) of TFs can weakly interact to stabilize TF chromatin binding^41^, it is possible that NPAT binds to Pol II via weak multivalent IDR interactions. To test this idea, we profiled NPAT chromatin binding using CUT&Tag and its association with transcription bubbles using NPAT-tethered KAS-CUT&Tag in K562 cells. We first examined four active and closely spaced bidirectional H2A/H2B histone gene pairs. Strikingly, KAS-CUT&Tag signals for NPAT and Pol II precisely co-localized and were stronger than their CUT&Tag signals (Fig. 7a). These high signals are consistent across all cell cycle phases, indicating that Pol II generates transcription bubbles at these histone genes and that NPAT must be colocalized with this Pol II throughout the cell cycle. By contrast, non-histone gene pairs exhibited much weaker signals than H2A/H2B genes with neither stronger NPAT and Pol II KAS-CUT&Tag signals relative to CUT&Tag nor precise NPAT–Pol II co-localization (Fig. 7b,c).

To assess NPAT–Pol II co-localization more comprehensively, we performed MACS2 peak calling on NPAT S-phase KAS-CUT&Tag data and identified 36 peaks overlapping the 5′ ends of histone gene clusters on chromosomes 1 and 6. Pol II KAS-CUT&Tag signals were centered over these peaks during S-phase, and both NPAT and Pol II KAS-CUT&Tag signals were markedly elevated relative to their respective CUT&Tag signals, indicating high levels of co-localized NPAT and Pol II at histone promoters (Fig. S8a). This association persisted throughout the cell cycle, with Pol II signals centered over NPAT peaks in both G1, S and S/G2 phases (Fig. 7c). In addition, NPAT and Pol II KAS-CUT&Tag signals showed concordant enrichment across RD-histone genes (Fig. 7d). These results indicate that NPAT is juxtaposed to transcription bubbles via interaction with Pol II at histone genes.

To assess NPAT–Pol II co-localization around the HLB, we selected the strongest 36 NPAT S-phase KAS-CUT&Tag peaks near HLBs that did not overlap RD-histone genes and the strongest 36 peaks located outside of HLBs as controls. We found that Pol II KAS-CUT&Tag and Pol II CUT&Tag signals were centered over the NPAT peaks near HLBs (Fig. S8b), implying that NPAT is also positioned adjacent to transcription bubbles via interaction with Pol II, even at non-histone gene sites. For the 36 peaks ouside of the HLB, 3 coincided with tandem AGGGTT repeats and were excluded. Of the remaining 33 peaks, Pol II KAS-CUT&Tag signals were centered over NPAT (Fig. S8c). Notably, 13 of these peaks were within 0.5 Mb of the major histone gene cluster *HIST1*, and 4 others were elsewhere on the 6p arm (Fig. S8c). Thus, 17 of the 33 euchromatic NPAT peaks (~52%) were located on chromosome 6p, even though this comprises only ~2% of the genome. This striking enrichment suggests that proximity to the HLB increases local NPAT concentration and binding, facilitating its positioning near transcription bubbles association through interactions with Pol II. Together, KAS-CUT&Tag profiling implies that NPAT chromatin binding arises from weak multivalent interactions with Pol II within or near HLBs, contributing to its high specificity.

## Discussion

KAS-CUT&Tag is a rapid, low-input, and easily standardized method, enabling direct and efficient mapping of transcription bubbles. While KAS-seq maps ssDNA and can be used to infer transcription bubble locations, its signals are not specific to bubbles formed by Pol II and may also arise from DNA damage, R-loops, non-canonical structures, or DNA replication^17^. KAS-ATAC-seq increases specificity by combining ssDNA labeling with Tn5 tagmentation but cannot resolve protein-specific interactions^19,20^. RNA-based methods, including RNA-seq^42^, GRO-seq^43,44^, PRO-seq^45^, NET-seq^46^, mNET-seq^47^, SLAM-seq^48^, and TimeLapse-seq^49^, indirectly infer Pol II dynamics via steady-state or nascent transcripts, often requiring high cell input and showing limited sensitivity to low-abundance RNAs.

Using an antibody raised against the unmodified Pol II CTD, KAS-CUT&Tag provides an unbiased and direct readout of transcription bubble dynamics across all cellular contexts. We found that inhibition of pause release increased bubble density at promoters, while blocking the reactivation of backtracked Pol II led to reduced bubble density at promoters and post-transcription end sites, demonstrating the sensitivity of Pol II KAS-CUT&Tag in detecting general transcriptional dynamics. Traditionally, extracellular signals are thought to regulate transcription via intracellular intermediates that activate transcription factors, which then regulate Pol II activity at target genes. However, recent studies have shown that nearly one-quarter of the ~500 human tyrosine kinases can directly phosphorylate the Pol II CTD at the Y1, T4, or S7 residues, with each kinase targeting distinct promoter subsets, likely via specific transcription factors interactions^50^. Detection of these modifications has been limited by poor antibody performance, due to the close proximity of the Y1, T4, and S7 residues to other phosphorylated positions. Pol II KAS-CUT&Tag overcomes this limitation by directly profiling transcription bubbles without relying on CTD phosphorylation, making it a powerful tool to investigate the effects of kinase and transcription factors perturbations. KAS-CUT&Tag is general, and should be adaptable to other eukaryotic and prokaryotic RNA polymerases. Additionally, KAS-CUT&Tag may be extended to specifically profile single-stranded regions during replication, recombination and repair with suitable antibodies.

KAS-CUT&Tag also enables detection of TFs and regulatory proteins juxtaposed to transcription bubbles through their association with Pol II. Because Pol II generates transcription bubbles, co-localization of NPAT and Pol II KAS-CUT&Tag signals indicates tight physical association. They precisely colocalize not only at histone genes, but also at non-genic sites within and near the HLB at two orders of magnitude above background. Live-cell imaging has shown that weak IDR heterotypic interactions stabilize TFs binding to chromatin^51^. As NPAT is mostly disordered and the Pol II CTD is fully disordered, a simple explanation for their co-localization in our experiments is that their juxtaposition is mediated by weak heterotypic interactions between their IDRs (Fig. 7e top panel). NPAT is phosphorylated by CDK2 to activate histone gene transcription at the G1/S transition^38^. *In vitro,* CDK2 phosphorylates the Pol II CTD at serine-2 with ~6-fold higher activity than canonical transcriptional kinases CDK9, CDK12, or CDK13^50^. Given that Pol II pause release depends on serine-2 phosphorylation, we suggest that dual phosphorylation by CDK2 at the G1/S transition introduces strong negative charges to both NPAT and the Pol II CTD. The bulk and electrostatic repulsion of so many phosphates may weaken NPAT-Pol II IDR interactions and promote the release of Pol II into productive elongation^52^ (Fig. 7e, bottom panel).

We have shown that, in the non-malignant GM12878 lymphoblastoid cell line, Pol II accumulates at HLBs during S phase relative to G1 and G2, whereas S-phase specificity is lost in the malignant leukemia K562 cell line (Fig. 6c). Furthermore, in K562 cells, Pol II remains engaged at transcription bubbles throughout interphase, and NPAT likewise remains juxtaposed to transcription bubbles at histone gene promoters (Fig. 7c). This persistent Pol II–NPAT association at transcription bubbles may facilitate G1-to-S transitions through CDK2-mediated histone gene activation. Consistent with this model, only 11% of K562 cells were in G1 and 66% were in S phase, whereas 39% of GM12878 cells were in G1 and 33% were in S phase. Given that histone gene overexpression^53^, and shortened total cell-cycle duration^54^ are CDK2-dependent hallmarks of cancer, our findings suggest that, in non-malignant cells, Pol II initiation at histone genes is normally restricted to S phase to tightly couple histone production to DNA replication, whereas in cancer cells, persistent Pol II–NPAT association at transcription bubbles throughout interphase may drive histone gene overexpression and accelerated cell-cycle progression.

## Methods

### Human cell culture

Human K562 (ATCC, CCL-243), HEK293T (ATCC, CRL-11268), and GM12878 (Coriell Institute) cells were cultured under standard conditions at 37 °C with 5% CO_2_ according to the manufacturer’s instructions. K562 cells were maintained in IMDM (ATCC, 30–2005) supplemented with 10% fetal bovine serum (FBS; HyClone, SH30070.03). HEK293T cells were grown in high-glucose DMEM (Gibco, 10566016) supplemented with 10% FBS and 1X Antibiotic-Antimycotic (Gibco, 15240062). GM12878 cells were maintained in RPMI-1640 with 2mM L-glutamine (Gibco, 11875093) supplemented with 15% FBS. For transcription inhibition experiments, inhibitors were first dissolved in DMSO, then diluted into the growth medium to the desired final concentrations and added directly to the cells. Transcription inhibitors included Triptolide (10 μM, Selleckchem, S3604), Flavopiridol hydrochloride hydrate (1 μM, Sigma-Aldrich, FL3055), and Actinomycin D (5 μg/mL, Sigma-Aldrich, A9415). DMSO (1:1,000 v/v) was used as a vehicle control. K562 cells were harvested 1 hour after treatment and processed for KAS-CUT&Tag profiling. All data in this study were generated from K562 cells, except for the WT and TFIIS KO^27^ samples, which are from HEK293T cells.

### Antibodies

The following primary antibodies were used: H3K36me3 (Rabbit monoclonal, Epicypher, 13–0058); Pol II (Mouse monoclonal, Sigma-Aldrich, 05–952-I); Pol II-Ser2p (Rabbit monoclonal, Cell Signaling Technology, 13499S); Pol II-Ser5p (Rabbit monoclonal, Cell Signaling Technology, 13523S); U2AF2 (Rabbit polyclonal, Abcam, ab37530); NPAT (Rabbit polyclonal, Invitrogen, PA565419) and NPAT (Mouse monoclonal, Abcam, ab307837). The following secondary antibodies were used: Guinea Pig anti-Rabbit IgG H&L (Antibodies Online, ABIN101961); Rabbit Anti-Mouse IgG H&L (Abcam, ab46540); Alexa Fluor^®^ 488 AffiniPure^®^ Fab Fragment Goat Anti-Mouse IgG (Jackson ImmunoResearch, 115–547-003) and Rhodamine Red^™^-X (RRX) AffiniPure^®^ Fab Fragment Goat Anti-Rabbit IgG (Jackson ImmunoResearch, 111–297-003). Final antibody concentrations used in CUT&Tag, KAS-CUT&Tag, CUT&RUN and Immunofluorescence staining experiments are provided in the corresponding protocol sections.

### Fluorescence-Activated Cell Sorting

To purify G1, S, G2 population of K562 cells, 10 million cells were resuspended in ice-cold harvest buffer (1x PBS with 2 mM EDTA, 2% FBS, 1 mM Spermidine, 0.05% Triton-X-100) and briefly fixed with 0.1% PFA for 2 minutes. Fixation was quenched with 2.5 M glycine for 5 minutes, then cells were spun down and resuspended in harvest buffer with 2.5 μM DRAQ5 (Thermo Scientific, 62251) to stain DNA. After at least 10 minutes of incubation, three cell cycle fractions were isolated on a BD Discover S8 imaging flow cytometer based on DRAQ5 intensity.

### Immunofluorescence staining

K562 and GM12878 cells were processed for immunofluorescence staining as follows. For EdU labeling, cells were incubated with 10 μM EdU in growth medium for 15 min before harvest. 500,000 cells were pelleted and resuspended in 500 μL of 4% formaldehyde (4% formaldehyde in 1× PBS; Thermo Scientific, 28906) and fixed on ice for 5 min. Fixation was quenched by addition of 25 μL of 2.5 M glycine, followed by centrifugation. Cells were then resuspended in 1 mL of PBST (1× PBS, 0.1% Triton X-100), pelleted, and resuspended again in 1 mL of 1× PBS. For slide preparation, 50 μL of cell suspension (approximately 50,000 cells) was deposited onto each glass slide by cytospin and immediately washed in 1× PBS in a Coplin jar. Cells were then blocked for 30 min at room temperature in 100 μL of wash blocking buffer (20 mM HEPES, pH 7.5, 150 mM NaCl, 0.5 mM spermidine, 0.1% Triton X-100, one EDTA-free protease inhibitor tablet per 50 mL (Sigma-Aldrich, 05056489001), 0.5% BSA (Sigma-Aldrich, A7906), and 0.5% casein (Sigma-Aldrich, C7078)). Rabbit anti-Pol II-Ser5P and mouse anti-NPAT primary antibodies were diluted 1:200 in antibody blocking buffer (wash blocking buffer supplemented with 2 mM EDTA) and pre-incubated for 30 min at 4°C. After blocking, 20 μL of the primary antibody dilution was added to each slide, and slides were incubated overnight at 4°C in a humid chamber. The following day, slides were washed in 1× PBS in a Coplin jar and blocked again for 30 min at room temperature in the same wash blocking buffer. Secondary antibodies (anti-rabbit Rhodamine and anti-mouse Fab-Alexa Fluor 488) were diluted 1:500 in wash blocking buffer and pre-incubated for 30 min at 4°C. 20 μL of the secondary antibody dilution was then added to each slide, followed by incubation for 1 h at 4°C. After secondary antibody incubation, slides were washed in 1× PBS and subjected to EdU detection using the Invitrogen Click-iT Plus EdU Cell Proliferation Kit with Alexa Fluor 647 dye (Fisher Scientific, C10640), according to the manufacturer’s instructions. The click reaction was carried out for 1 h at room temperature protected from light, followed by two washes in 1× PBS. Nuclear DNA was stained with 1× DAPI for 5 min at room temperature. Slides were then washed in 1× PBS, mounted in 80% glycerol, coverslipped, and sealed with nail polish before imaging.

### Fluorescence image acquisition and analysis

Images were acquired using a Leica Stellaris 8 scanning confocal microscope equipped with a 63×/1.4 NA oil HC PL APO CS2 objective. Z-stacks were collected from K562 and GM12878 cells. DAPI, Alexa Fluor 488, Rhodamine, and Alexa Fluor 647 were excited with 405 nm, 495 nm, 571 nm, and 649 nm lasers, respectively, and detected at 429–502 nm, 503–567 nm, 579–646 nm, and 654–783 nm, respectively. Images were analyzed using Imaris v11.0.1 for cell segmentation and detection of Pol II-Ser5P and NPAT foci, and the number of foci per cell was quantified using Imaris Cell. Colocalized foci were defined as Pol II-Ser5P foci with NPAT mean intensity greater than 30. The numbers of Pol II-Ser5P foci, NPAT foci, and colocalized foci per cell are reported in Supplementary Table 1. EdU staining patterns were used to classify cells as early, mid, or late S phase. Among EdU-negative cells, those with fewer than 9 NPAT foci in K562 or fewer than 2 NPAT foci in GM12878 were classified as G1, and the remaining interphase cells were classified as G2. Pseudocolored maximum-intensity projections were generated from z-stacks. For display, identical minimum and maximum intensity settings were applied to all images within each cell type and channel, and images were composited in Fiji. Images exported from Fiji were assembled into the final figures using Adobe Illustrator.

### CUT&RUN profiling

CUT&RUN was carried out following a previously described protocol^55^, with several modifications. A total of 600,000 cells were collected by centrifugation and resuspended in wash buffer (20 mM HEPES pH 7.5, 150 mM NaCl, 0.5 mM spermidine, and one EDTA-free protease inhibitor tablet per 50mL (Sigma-Aldrich, 05056489001)). The cell suspension was then incubated with activated ConA-coated magnetic beads (5 μl per sample; Bangs Laboratories, BP531) for 10 minutes at room temperature to allow bead binding. Following this, the bead-bound cells were aliquoted into individual PCR tubes, the supernatant was removed, and the beads were resuspended in 50 μl of antibody buffer (wash buffer containing 0.01% Digitonin and 2 mM EDTA). Primary antibody against U2AF2 were added at a 1:20 dilution, and samples were incubated overnight at 4 °C. After antibody binding, the samples were washed twice with dig wash buffer (wash buffer + 0.01% Digitonin). They were then incubated for 10 minutes at room temperature with Protein A–MNase (pA-MN, prepared in-house) at a 1:130 dilution, followed by two additional washes with dig wash buffer. To initiate MNase digestion, the cell-bead slurry was resuspended in dig wash buffer containing 2 mM CaCl_2_ and incubated at 4 °C for 2 hours. The digestion reaction was stopped by adding 33 μl of stop buffer (340 mM NaCl, 20 mM EDTA, 4 mM EGTA, 50 μg/ml glycogen (Sigma-Aldrich, 10930193001), 50 μg/ml RNase A (Thermo Fisher Scientific, EN0531), and 2 pg/ml *E. coli* spike-in DNA), followed by a 10-minute incubation at 37 °C to release DNA fragments. The supernatant was collected, and DNA was purified using the CUTANA^™^ DNA Purification Kit (EpiCypher, 14–0050) according to the manufacturer’s instructions. The resulting DNA was then used for library preparation as described previously^55^.

### CUT&Tag profiling

We performed CUT&Tag based on the CUT&Tag-direct protocol^56,57^ with slight modifications. To begin, 50,000 cells were pelleted and gently resuspended in wash buffer (20 mM HEPES pH 7.5, 150 mM NaCl, 0.5 mM spermidine, 0.05% Triton X-100, one EDTA-free protease inhibitor tablet per 50mL (Sigma-Aldrich, 05056489001)). ConA-coated magnetic beads (5 μl per sample; Bangs Laboratories, BP531) were pre-washed and activated in beads binding buffer (20 mM HEPES pH 7.9, 10 mM KCl, 1 mM CaCl_2_, 1 mM MnCl_2_), then added to the cell suspension and incubated at room temperature for 10 minutes. Bead-bound cells were transferred to antibody binding buffer (wash buffer + 2 mM EDTA), split into 0.5 ml tubes, and incubated overnight at 4 °C with primary antibody against H3K36me3 or Pol II-Ser5p (1:10 dilution). Following incubation, unbound antibody was removed by washing with wash buffer, and bead-bound cells were subsequently incubated in wash buffer containing secondary antibody (1:15 dilution) for 1 hour at 4 °C. After another wash with wash buffer, beads were resuspended in 300-wash buffer (wash buffer + 150 mM NaCl) containing Protein A/G-Tn5 (1:10 dilution; EpiCypher, 15–1117) and incubated for 1 hour at 4 °C. Unbound pAG-Tn5 was removed by washing using 300-wash buffer, and tagmentation was performed by resuspending beads in and incubating at 37 °C for 1 hour. Following tagmentation, beads were washed with TAPS buffer (10 mM TAPS, 0.2 mM EDTA) and resuspended in release solution (5 μl total; 10 mM TAPS, 0.1% SDS, and 1:10 Thermolabile Proteinase K; New England Biolabs, P8111S). Samples were incubated in a thermocycler with a heated lid at 37 °C for 1 hour, then at 58 °C for an additional hour. SDS was quenched by adding 4 μl of 1.5% Triton X-100. For library amplification, 2 μl each of barcoded 10 μM i5 and i7 primers were combined with 42 μl of PCR master mix (10 μl HiFi buffer, 1.5 μl 10 mM dNTPs, 1 μl KAPA HiFi polymerase, and 29.5 μl H_2_O; Roche, 07958846001). PCR cycling conditions were: 58 °C for 5 min; 72 °C for 5 min; 98 °C for 30 s; followed by 12 cycles of 98 °C for 10 s and 60 °C for 10 s; then 72 °C for 1 min and hold at 12 °C. Libraries were purified using HighPrep paramagnetic beads (MagBio, AC-60500) at a 1.3:1 bead-to-sample volume ratio.

### KAS-CUT&Tag profiling

A total of 30,000 cells were pelleted and gently resuspended in antibody binding buffer (20 mM HEPES pH 7.5, 150 mM NaCl, 0.5 mM spermidine, 0.05% Triton X-100, 2 mM EDTA, and EDTA-free protease inhibitor tablet). Separately, 5 μl of ConA-coated magnetic beads (Bangs Laboratories, BP531) were washed and activated in beads binding buffer (20 mM HEPES pH 7.9, 10 mM KCl, 1 mM CaCl_2,_ 1 mM MnCl_2_), then added to the cell suspension and incubated for 10 minutes at room temperature. After binding, bead-bound cells were transferred to fresh antibody binding buffer, divided into 0.5 ml tubes, and incubated with 100 mM N_3_-kethoxal (ApexBio, A8793) in 1× PBS at 37 °C for 10 minutes. Cells were subsequently washed twice in DPBS and fixed in 0.1% formaldehyde (Thermo Scientific, 28906) for 1 minute. Following fixation, cells were rinsed in wash buffer (20 mM HEPES pH 7.5, 150 mM NaCl, 0.5 mM spermidine, 0.05% Triton X-100, one EDTA-free protease inhibitor tablet per 50mL (Sigma-Aldrich, 05056489001)) and incubated overnight at 4 °C with primary antibody against Pol II, Pol II-Ser5p, Pol II-Ser2p or NPAT (1:10 dilution). The next day, cells were washed to remove unbound antibody and incubated with secondary antibody (1:15 dilution in wash buffer) for 1 h at 4 °C. After washing by wash buffer, beads were resuspended in 300-wash buffer (wash buffer + 50 mM NaCl) containing pAG-Tn5 transposase (1:10 dilution; EpiCypher, 15–1117) and incubated at 4 °C for 1 h. Excess pAG-Tn5 was removed by washing with 300-wash buffer, and tagmentation was performed by incubating in CUTAC-DMF tagmentation buffer (20% N,N-dimethylformamide (Sigma-Aldrich, D8654), 10 mM TAPS PH8.5, 5 mM, 0.05% Triton X-100, 5 mM MgCl_2_) at 37 °C for 1 h. Following tagmentation, beads were washed with TAPS buffer (10 mM TAPS, 0.2 mM EDTA) and resuspended in release solution (5 μl total; 10 mM TAPS, 0.1% SDS, and 1:10 Thermolabile Proteinase K(New England Biolabs, P8111S). Samples were incubated at 37 °C for 1 h, followed by 58 °C for 1 h in a thermocycler with a heated lid. SDS was quenched by adding 4 μl of 1.5% Triton X-100. DNA was purified using the DNA Clean & Concentrator-5 Kit (Zymo Research, D4014) and eluted in 10 μl of 25 mM K_3_BO_3_ (pH 7.0). For biotinylation, the 10 μl DNA eluate was mixed with 34.5 μl of 25 mM K_3_BO_3_ (pH 7.0), 5 μl of 10× PBS, and 2.5 μl of 20 mM DBCO-PEG_4_-Biotin (Sigma, 760749), and incubated at 37 °C for 1.5 hr. Biotinylated DNA was purified again with the Zymo kit and eluted in 55 μl of 25 mM K_3_BO_3_ (pH 7.0). A 5 μl aliquot was reserved to prepare the CUT&Tag input library; the remaining 50 μl biotinylated DNA was used for streptavidin-based pull-down. For immunoprecipitation, 5 μl of Dynabeads MyOne Streptavidin T1 (Thermo Fisher, 65602) or Dynabeads MyOne Streptavidin C1 (Thermo Fisher, 65001) were pre-washed with 1× B&W buffer and resuspended in 50 μl of 2× B&W buffer (10 mM Tris-HCl pH 7.4, 1 mM EDTA, 2 M NaCl, 0.1% Tween-20). Pre-washed Dynabeads were added to the biotinylated DNA and incubated for 15 min at room temperature. Beads were then washed five times with 1× B&W buffer and resuspended in 28 μl nuclease-free water for library preparation. For the input sample, 23 μl of nuclease-free water was added to 5 μl of DNA. For PCR amplification, each 22 μl reaction contained 2 μl each of barcoded 10 μM i5 and i7 primers, and 18 μl of PCR master mix (10 μl HiFi buffer, 1.5 μl 10 mM dNTPs, 1 μl KAPA HiFi polymerase, 5.5 μl H_2_O; Roche, 07958846001). Cycling conditions were: 58 °C for 5 min, 72 °C for 5 min, 95 °C for 10 min; followed by 12 cycles of 98 °C for 10 s and 60 °C for 10 s; then 72 °C for 1 min, ending with a 12 °C hold. Final libraries were purified using HighPrep paramagnetic beads (MagBio, AC-60500) at a 1.3:1 bead-to-sample ratio.

A step-by-step protocol is available from Protocols.io: DOI: 10.17504/protocols.io.e6nvwn82dvmk/v1

### DNA sequencing and data processing

Library size distributions and molar concentrations were assessed using an Agilent 4200 TapeStation. Libraries were pooled to ensure equal representation and adjusted to the final concentration recommended by the manufacturer. Paired-end 50 × 50 bp sequencing was performed on an Illumina NovaSeq X Plus platform by the Fred Hutchinson Cancer Center Genomics Shared Resources. Sequencing data were processed as described. (https://www.protocols.io/view/cut-amp-tag-data-processing-and-analysis-tutorial-e6nvw93x7gmk/v1). For processing sequencing data, adapter sequences were trimmed from 50 bp paired-end reads using Cutadapt 4.4^58^ with the specified parameters “-j 8 --nextseq-trim 20 -m 20 -a AGATCGGAAGAGCACACGTCTGAACTCCAGTCA -A AGATCGGAAGAGCGTCGTGTAGGGAAAGAGTGT -Z”

We used Bowtie2 2.5.1^59^ to map the paired-end 50 bp reads to the hg19 human genome reference sequence from UCSC with parameters “--very-sensitive-local --soft-clipped-unmapped-tlen --dovetail --no-mixed --no-discordant -q --phred33 -I 10 -X 1000”. MACS2 peak calling for NPAT KAS-CnT data was performed using macs2 callpeak -t $bed -f BEDPE -g hs --keep-dup all -p 1e-10 -n $sname.

### Data analysis and visualization

BedGraph files were generated using BEDTools^60^ genomecov, and subsequently converted to bigWig format with bedGraphToBigWig. The resulting bigWig files were coverage-normalized by scaling read counts at each base pair to the total size of the reference genome (hg19; 3,095,693,983 bp), such that a uniformly distributed signal would yield a value of 1 across all positions. This normalization enables the identification of regions with enriched protein epitope signals on DNA. Genomic bigWig tracks were visualized using the Integrated Genome Viewer (IGV). Heatmaps were produced using deepTools (version 3.5.1)^61^ with the computeMatrix and plotHeatmap functions. H3K36me3 CUT&Tag (GSM8343606, GSM8343607) and U2AF2 CUT&RUN (GSM8339598–GSM8339602) bigWig files aligned to hg19 in K562 cells were downloaded from GEO^36^. The KAS-seq bigWig file mapped to hg38 from K562 cells was downloaded from GEO (GSM6219605)^62^ and reprocessed to generate hg19-mapped bigWig files for use in this study. Paired-end FASTQ files for standard ATAC-seq (SRR10319911, SRR10319912)^18^ and N_3_-kethoxal-treated ATAC-seq (SRR28028139^20^, SRR28028140^20^, SRR28764289^19^) from HEK293T cells were downloaded from the SRA and aligned to the hg19 human reference genome using Bowtie2 (v2.5.1)^59^. SAM files were converted to coordinate-sorted BAM files using SAMtools^63^. BED files representing ATAC-seq fragments were generated from the BAM files using BEDTools (v2.30.0)^60^ for downstream analysis. To generate V-plots, fragment length was plotted as a function of the distance from each fragment’s midpoint to the TSS of 12,397 RefSeq-annotated protein-coding genes. 12 bidirectional non-histone gene pairs were obtained from Additional File 1 of Wang et al^64^.

## Supplementary Material

Supplement 1

Supplement 2

## Data Availability

All primary sequence data and interpreted track files for sequence data generated in this study have been deposited at the Gene Expression Omnibus: GSE317877 To review GEO accession GSE317877, go to: https://urldefense.com/v3/__https://www.ncbi.nlm.nih.gov/geo/query/acc.cgi?acc=GSE317877__;!!GuAItXPztq0!hY23CqdCgAnJ_wP5hJ2qtcg7pbG7qD9JHpe1JEEEV9CwCTSrOOyseqBVTu01CZgk_nJVaIY3D2ouxIul$ Enter token shqzkmwwxrwzfqt into the box A step-by-step protocol is available to reviewers from a Protocols.io Reserve link for anonymous access: https://www.protocols.io/private/4D806056FC0A11F0A4600A58A9FEAC02

## Code availability

This paper does not report original code. Any additional information required to reanalyze the data reported in this paper is available upon request.

## References

[1] Malik S. & Roeder R. G. Regulation of the RNA polymerase II pre-initiation complex by its associated coactivators. Nature Reviews Genetics 24, 767–782 (2023). 10.1038/s41576-023-00630-9 PMC11088444 37532915

[2] Fishburn J., Tomko E., Galburt E. & Hahn S. Double-stranded DNA translocase activity of transcription factor TFIIH and the mechanism of RNA polymerase II open complex formation. Proceedings of the National Academy of Sciences 112, 3961–3966 (2015). 10.1073/pnas.1417709112 PMC4386358 25775526

[3] Schilbach S., Aibara S., Dienemann C., Grabbe F. & Cramer P. Structure of RNA polymerase II pre-initiation complex at 2.9 Å defines initial DNA opening. Cell 184, 4064–4072. e4028 (2021). 34133942 10.1016/j.cell.2021.05.012

[4] Chen X. Structural visualization of transcription initiation in action. Science 382, eadi5120 (2023). 10.1126/science.adi5120 38127763

[5] Jeronimo C. & Robert F. Kin28 regulates the transient association of Mediator with core promoters. Nature Structural & Molecular Biology 21, 449–455 (2014). 10.1038/nsmb.2810 PMC3997488 24704787

[6] Wong Koon H., Jin, Y. & Struhl, K. TFIIH Phosphorylation of the Pol II CTD Stimulates Mediator Dissociation from the Preinitiation Complex and Promoter Escape. Molecular Cell 54, 601–612 (2014). 10.1016/j.molcel.2014.03.024 24746699 PMC4035452

[7] Velychko T. CDK7 kinase activity promotes RNA polymerase II promoter escape by facilitating initiation factor release. Molecular Cell 84, 2287–2303.e2210 (2024). 10.1016/j.molcel.2024.05.007 38821049

[8] Vos S. M., Farnung L., Urlaub H. & Cramer P. Structure of paused transcription complex Pol II–DSIF–NELF. Nature 560, 601–606 (2018). 10.1038/s41586-018-0442-2 30135580 PMC6245578

[9] Adelman K. & Lis J. T. Promoter-proximal pausing of RNA polymerase II: emerging roles in metazoans. Nature Reviews Genetics 13, 720–731 (2012). 10.1038/nrg3293 PMC3552498 22986266

[10] Garriga J. & Graña X. Cellular control of gene expression by T-type cyclin/CDK9 complexes. Gene 337, 15–23 (2004). 10.1016/j.gene.2004.05.007 15276198

[11] Vos S. M. Structure of activated transcription complex Pol II–DSIF–PAF–SPT6. Nature 560, 607–612 (2018). 30135578 10.1038/s41586-018-0440-4

[12] Porrua O. & Libri D. Transcription termination and the control of the transcriptome: why, where and how to stop. Nature Reviews Molecular Cell Biology 16, 190–202 (2015). 10.1038/nrm3943 25650800

[13] Proudfoot N. J. Transcriptional termination in mammals: Stopping the RNA polymerase II juggernaut. Science 352, aad9926 (2016). 10.1126/science.aad9926 27284201 PMC5144996

[14] Kettenberger H., Armache K.-J. & Cramer P. Complete RNA Polymerase II Elongation Complex Structure and Its Interactions with NTP and TFIIS. Molecular Cell 16, 955–965 (2004). 10.1016/j.molcel.2004.11.040 15610738

[15] Barnes C. O. Crystal structure of a transcribing RNA polymerase II complex reveals a complete transcription bubble. Molecular cell 59, 258–269 (2015). 26186291 10.1016/j.molcel.2015.06.034 PMC4643057

[16] Noe Gonzalez M., Blears D. & Svejstrup J. Q. Causes and consequences of RNA polymerase II stalling during transcript elongation. Nature Reviews Molecular Cell Biology 22, 3–21 (2021). 10.1038/s41580-020-00308-8 33208928

[17] Wu T., Lyu R., You Q. & He C. Kethoxal-assisted single-stranded DNA sequencing captures global transcription dynamics and enhancer activity in situ. Nature Methods 17, 515–523 (2020). 10.1038/s41592-020-0797-9 32251394 PMC7205578

[18] Tak Y. E. Augmenting and directing long-range CRISPR-mediated activation in human cells. Nat Methods 18, 1075–1081 (2021). 10.1038/s41592-021-01224-1 34354266 PMC8446310

[19] Kim S. H., Marinov G. K. & Greenleaf W. J. KAS-ATAC reveals the genome-wide single-stranded accessible chromatin landscape of the human genome. bioRxiv, 2024.2005.2006.591268 (2024). 10.1101/2024.05.06.591268 PMC11789636 39572230

[20] Lyu R. Quantitative analysis of cis-regulatory elements in transcription with KAS-ATAC-seq. bioRxiv, 2024.2002.2029.582869 (2024). 10.1101/2024.02.29.582869 PMC11316786 39127768

[21] Titov D. V. XPB, a subunit of TFIIH, is a target of the natural product triptolide. Nature Chemical Biology 7, 182–188 (2011). 10.1038/nchembio.522 21278739 PMC3622543

[22] Sedlacek H. H. Mechanisms of action of flavopiridol. Critical Reviews in Oncology/Hematology 38, 139–170 (2001). 10.1016/S1040-8428(00)00124-4 11311660

[23] Sobell H. M. Actinomycin and DNA transcription. Proceedings of the National Academy of Sciences 82, 5328–5331 (1985). 10.1073/pnas.82.16.5328 PMC390561 2410919

[24] Kettenberger H., Armache K.-J. & Cramer P. Architecture of the RNA polymerase II-TFIIS complex and implications for mRNA cleavage. Cell 114, 347–357 (2003). 12914699 10.1016/s0092-8674(03)00598-1

[25] Schweikhard V. Transcription factors TFIIF and TFIIS promote transcript elongation by RNA polymerase II by synergistic and independent mechanisms. Proceedings of the National Academy of Sciences 111, 6642–6647 (2014). 10.1073/pnas.1405181111 PMC4020062 24733897

[26] Cheung A. C. & Cramer P. Structural basis of RNA polymerase II backtracking, arrest and reactivation. Nature 471, 249–253 (2011). 21346759 10.1038/nature09785

[27] Yang K. B. Persistence of backtracking by human RNA polymerase II. Molecular Cell 84, 897–909.e894 (2024). 10.1016/j.molcel.2024.01.019 38340716

[28] Nudler E. RNA polymerase backtracking in gene regulation and genome instability. Cell 149, 1438–1445 (2012). 10.1016/j.cell.2012.06.003 22726433 PMC3815583

[29] Adelman K. Efficient release from promoter-proximal stall sites requires transcript cleavage factor TFIIS. Molecular cell 17, 103–112 (2005). 15629721 10.1016/j.molcel.2004.11.028

[30] De Almeida S. F. Splicing enhances recruitment of methyltransferase HYPB/Setd2 and methylation of histone H3 Lys36. Nature structural & molecular biology 18, 977–983 (2011). 10.1038/nsmb.2123 21792193

[31] Kolasinska-Zwierz P. Differential chromatin marking of introns and expressed exons by H3K36me3. Nature genetics 41, 376–381 (2009). 19182803 10.1038/ng.322 PMC2648722

[32] Edmunds J. W., Mahadevan L. C. & Clayton A. L. Dynamic histone H3 methylation during gene induction: HYPB/Setd2 mediates all H3K36 trimethylation. The EMBO Journal 27, 406–420-420 (2008). 10.1038/sj.emboj.7601967 18157086 PMC2168397

[33] David C. J., Boyne A. R., Millhouse S. R. & Manley J. L. The RNA polymerase II C-terminal domain promotes splicing activation through recruitment of a U2AF65–Prp19 complex. Genes & development 25, 972–983 (2011). 21536736 10.1101/gad.2038011 PMC3084030

[34] Li B., Howe L., Anderson S., Yates J. R. & Workman J. L. The Set2 histone methyltransferase functions through the phosphorylated carboxyl-terminal domain of RNA polymerase II. Journal of Biological Chemistry 278, 8897–8903 (2003). 12511561 10.1074/jbc.M212134200

[35] Markert J. W., Soffers J. H. & Farnung L. Structural basis of H3K36 trimethylation by SETD2 during chromatin transcription. Science 387, 528–533 (2025). 10.1126/science.adn6319 39666822 PMC12366524

[36] Wu W., Ahmad K. & Henikoff S. Chromatin-bound U2AF2 splicing factor ensures exon inclusion. Molecular Cell (2025). 10.1016/j.molcel.2025.04.013 PMC13075997 40315850

[37] Kemp J. P. Cell cycle-regulated transcriptional pausing of Drosophila replication-dependent histone genes. bioRxiv, 2024.2012.2016.628706 (2024). 10.1101/2024.12.16.628706 PMC12260174 40397569

[38] Armstrong C., Passanisi V. J., Ashraf H. M. & Spencer S. L. Cyclin E/CDK2 and feedback from soluble histone protein regulate the S phase burst of histone biosynthesis. Cell Reports 42, 112768 (2023). 10.1016/j.celrep.2023.112768 37428633 PMC10440735

[39] Ahmad K. Cell-cycle-dependent repression of histone gene transcription by histone H4. bioRxiv, 2024.2012.2023.630206 (2025). 10.1101/2024.12.23.630206 PMC12819152 41491041

[40] Xu S. B. KPNA3 regulates histone locus body formation by modulating condensation and nuclear import of NPAT. J Cell Biol 224 (2025). 10.1083/jcb.202401036 PMC11613458 39621428

[41] Abidi A. A. Unstructured transcription factor interactions enable emergent specificity. Science 0, eaeb6487 10.1126/science.aeb6487 PMC13581189 41855276

[42] Wang Z., Gerstein M. & Snyder M. RNA-Seq: a revolutionary tool for transcriptomics. Nat Rev Genet 10, 57–63 (2009). 10.1038/nrg2484 19015660 PMC2949280

[43] Mahat D. B. Single-cell nascent RNA sequencing unveils coordinated global transcription. Nature (2024). 10.1038/s41586-024-07517-7 PMC11222150 38839954

[44] Core L. J., Waterfall J. J. & Lis J. T. Nascent RNA sequencing reveals widespread pausing and divergent initiation at human promoters. Science 322, 1845–1848 (2008). 19056941 10.1126/science.1162228 PMC2833333

[45] Mahat D. B. Base-pair-resolution genome-wide mapping of active RNA polymerases using precision nuclear run-on (PRO-seq). Nature protocols 11, 1455–1476 (2016). 27442863 10.1038/nprot.2016.086 PMC5502525

[46] Churchman L. S. & Weissman J. S. Nascent transcript sequencing visualizes transcription at nucleotide resolution. Nature 469, 368–373 (2011). 10.1038/nature09652 21248844 PMC3880149

[47] Nojima T. Mammalian NET-seq reveals genome-wide nascent transcription coupled to RNA processing. Cell 161, 526–540 (2015). 25910207 10.1016/j.cell.2015.03.027 PMC4410947

[48] Herzog V. A. Thiol-linked alkylation of RNA to assess expression dynamics. Nature Methods 14, 1198–1204 (2017). 10.1038/nmeth.4435 28945705 PMC5712218

[49] Schofield J. A., Duffy E. E., Kiefer L., Sullivan M. C. & Simon M. D. TimeLapse-seq: adding a temporal dimension to RNA sequencing through nucleoside recoding. Nature Methods 15, 221–225 (2018). 10.1038/nmeth.4582 29355846 PMC5831505

[50] Dabas P. Direct targeting and regulation of RNA polymerase II by cell signaling kinases. Science 390, eads7152 (2025). 10.1126/science.ads7152 41197002

[51] Abidi A. A., Dailey G. M., Tjian R. & G W Graham T. Collective unstructured interactions drive chromatin binding of transcription factors. bioRxiv, 2025.2005.2016.654615 (2025). 10.1101/2025.05.16.654615

[52] Henikoff S. & Hahn S. RNA polymerase II: the elephant in the room. Trends in Genetics (2026). 10.1016/j.tig.2026.01.008 PMC13242782 41820080

[53] Henikoff S. RNA polymerase II at histone genes predicts outcome in human cancer. Science 387, 737–743 (2025). 10.1126/science.ads2169 39946483 PMC12184985

[54] Chen D. Cell cycle duration determines oncogenic transformation capacity. Nature 641, 1309–1318 (2025). 10.1038/s41586-025-08935-x 40307557 PMC12119354

[55] Skene P. J. & Henikoff S. An efficient targeted nuclease strategy for high-resolution mapping of DNA binding sites. eLife 6, e21856 (2017). 10.7554/eLife.21856 28079019 PMC5310842

[56] Janssens D. H. Automated CUT&Tag profiling of chromatin heterogeneity in mixed-lineage leukemia. Nature Genetics 53, 1586–1596 (2021). 10.1038/s41588-021-00941-9 34663924 PMC8571097

[57] Kaya-Okur H. S., Janssens D. H., Henikoff J. G., Ahmad K. & Henikoff S. Efficient low-cost chromatin profiling with CUT&Tag. Nature protocols 15, 3264–3283 (2020). 32913232 10.1038/s41596-020-0373-x PMC8318778

[58] Martin M. Cutadapt removes adapter sequences from high-throughput sequencing reads. EMBnet. journal 17, 10–12 (2011).

[59] Langmead B. & Salzberg S. L. Fast gapped-read alignment with Bowtie 2. Nature methods 9, 357–359 (2012). 22388286 10.1038/nmeth.1923 PMC3322381

[60] Quinlan A. R. & Hall I. M. BEDTools: a flexible suite of utilities for comparing genomic features. Bioinformatics 26, 841–842 (2010). 20110278 10.1093/bioinformatics/btq033 PMC2832824

[61] Ramírez F. deepTools2: a next generation web server for deep-sequencing data analysis. Nucleic acids research 44, W160 (2016). 27079975 10.1093/nar/gkw257 PMC4987876

[62] Dou X. RBFOX2 recognizes N(6)-methyladenosine to suppress transcription and block myeloid leukaemia differentiation. Nat Cell Biol 25, 1359–1368 (2023). 10.1038/s41556-023-01213-w 37640841 PMC10495261

[63] Li H. The sequence alignment/map format and SAMtools. bioinformatics 25, 2078–2079 (2009). 19505943 10.1093/bioinformatics/btp352 PMC2723002

[64] Wang G. Identification of regulatory regions of bidirectional genes in cervical cancer. BMC medical genomics 6, S5 (2013). 10.1186/1755-8794-6-S1-S5 PMC3552671 23369456

[65] Lyu R. Quantitative analysis of cis-regulatory elements in transcription with KAS-ATAC-seq. Nature Communications 15, 6852 (2024). 10.1038/s41467-024-50680-8 PMC11316786 39127768

[66] Schraivogel D. High-speed fluorescence image–enabled cell sorting. Science 375, 315–320 (2022). 10.1126/science.abj3013 35050652 PMC7613231

