# Supplementary material for: KAS-CUT&Tag for direct mapping of transcription bubbles
Source: bioRxiv. 2026 May 19:2026.05.15.725569. Preprint. [Version 1] doi: 10.64898/2026.05.15.725569 (PMC13228418; doi:10.64898/2026.05.15.725569)
Supplement: Supplement 2 [file NIHPP2026.05.15.725569v1-supplement-2.pdf]

bioRxiv preprint doi: https://doi.org/10.64898/2026.05.15.725569; this version posted May 19, 2026. The copyright holder for this preprint (which was not certified by peer review) is the author/funder, who has granted bioRxiv a license to display the preprint in perpetuity. It is made available under a CC-BY 4.0 International license.

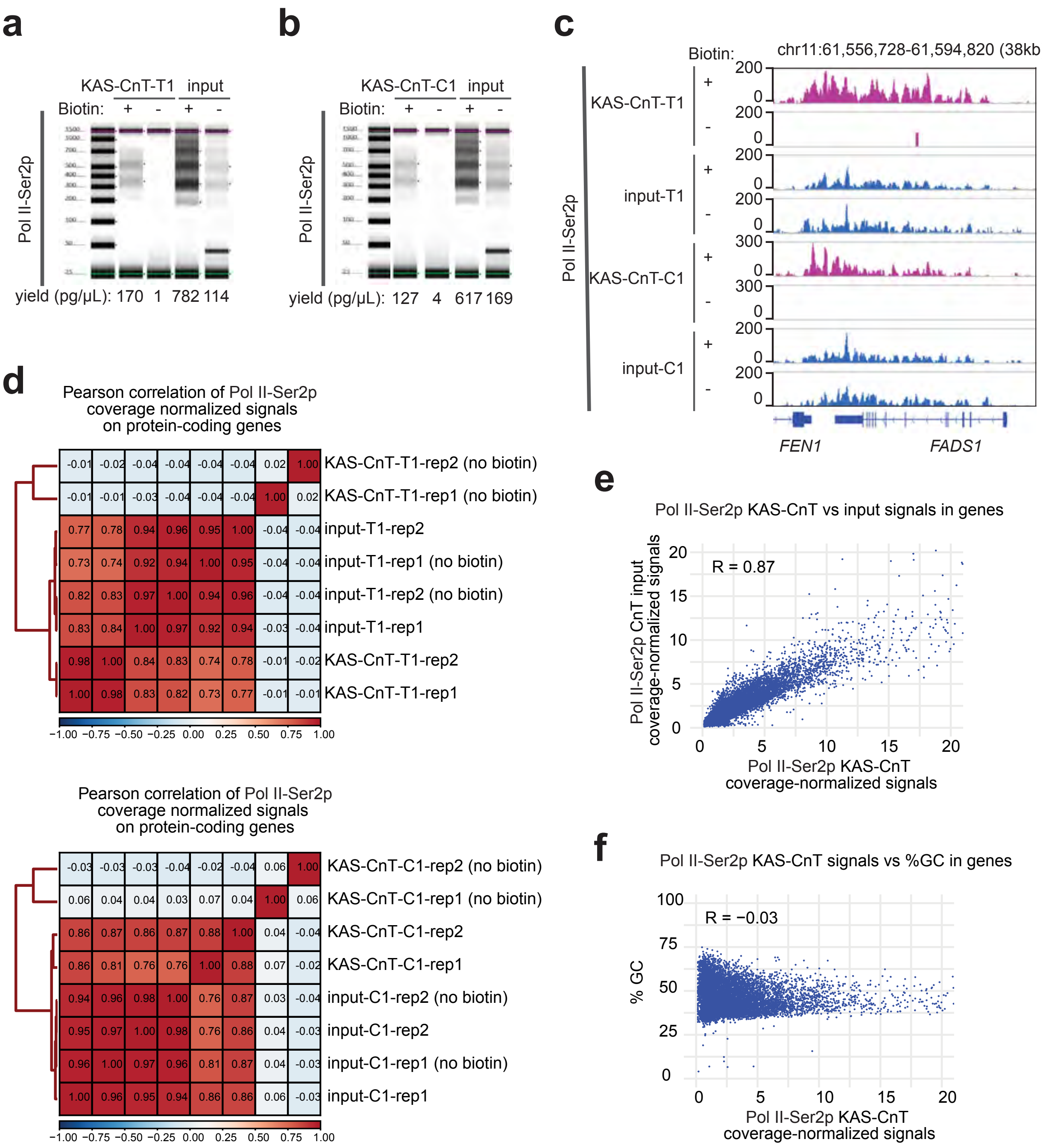

Fig.S1: Specificity and reproducibility of KAS-CUT&Tag.
a, b, Tapestation profiles of Pol II-Ser2P KAS-CnT and CnT input, with or without biotin click chemistry, in K562 cells. KAS-CnT-T1 and KAS-CnT-C1 refer to libraries enriched using Streptavidin T1 and C1 beads, respectively. **c,** Coverage-normalized signals for KAS-CnT and CnT input, with or without biotin click chemistry in K562. Input-T1 and Input-C1 are the corresponding input controls for KAS-CnT-T1 and KAS-CnT-C1, respectively. **d,** Pearson correlation of Pol II-Ser2P coverage-normalized signals across protein-coding genes. **e,** Scatter plot showing the correlation between Pol II-Ser2P KAS-CnT and CnT input coverage-normalized signals at protein-coding genes in K562. Each dot represents one gene. **f,** Scatter plot showing the relationship between Pol II-Ser2P KAS-CnT signal and GC content (%GC) across protein-coding genes in K562. Each dot represents one gene.

bioRxiv preprint doi: https://doi.org/10.64898/2026.05.15.725569; this version posted May 19, 2026. The copyright holder for this preprint (which was not certified by peer review) is the author/funder, who has granted bioRxiv a license to display the preprint in perpetuity. It is made available under a CC-BY 4.0 International license.

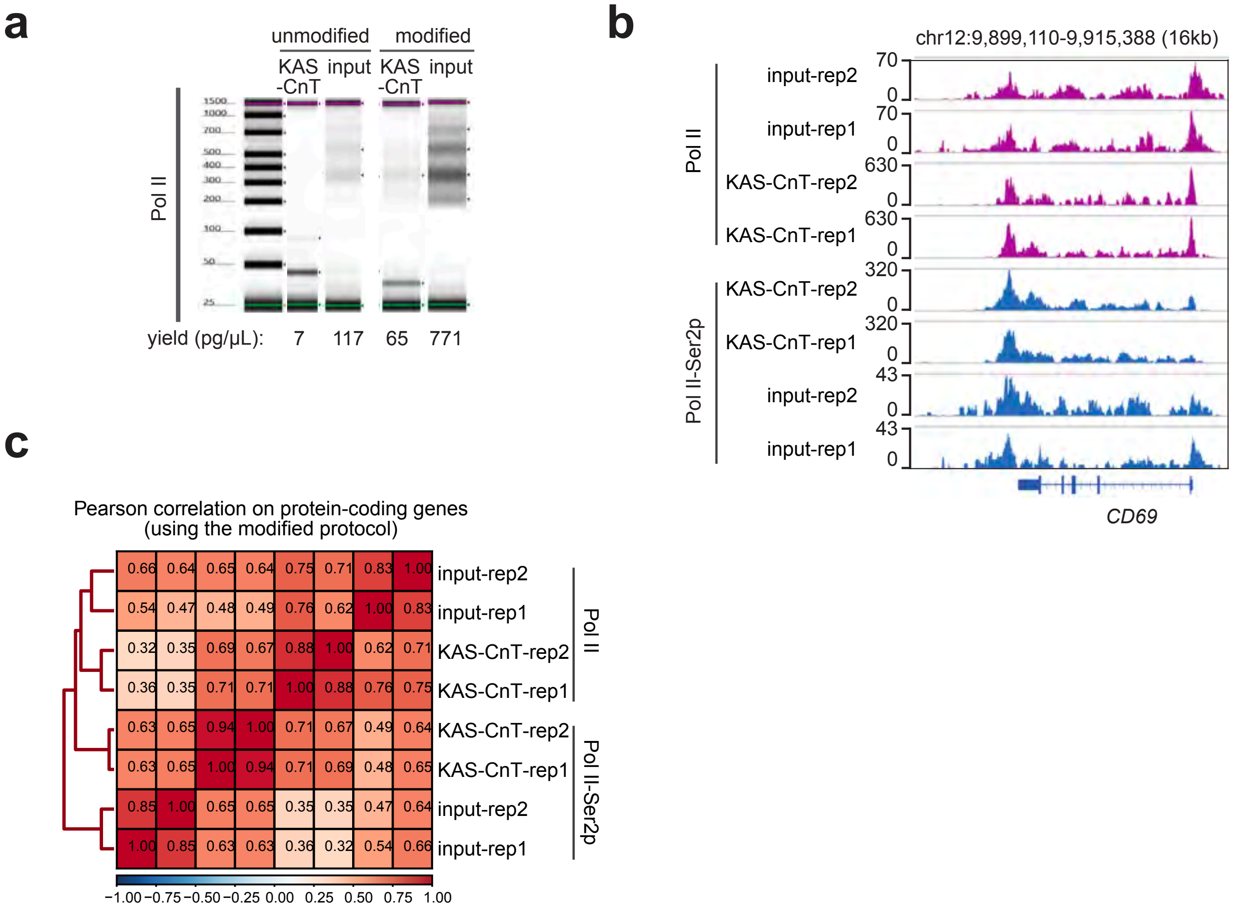

Fig.S2: Light crosslinking and CUTAC tagmentation increase KAS-CUT&Tag library yield while maintaining high reproducibility.

a, Tapestation profiles of Pol II KAS-CnT and input libraries in K562 cells. The modified protocol includes light crosslinking (0.1% formaldehyde for 1 min) after N_3-kethoxal treatment, followed by tagmentation under low-salt CUTAC conditions. The unmodified protocol omits crosslinking and uses tagmentation under 300 mM NaCl conditions. **b,** Coverage-normalized signals for Pol II and Pol II-Ser2P KAS-CnT, along with their respective input controls, in K562 cells generated using the modified protocol. **c,** Pearson correlation of Pol II-Ser2P coverage-normalized signals across protein-coding genes using the modified protocol.

bioRxiv preprint doi: https://doi.org/10.64898/2026.05.15.725569; this version posted May 19, 2026. The copyright holder for this preprint (which was not certified by peer review) is the author/funder, who has granted bioRxiv a license to display the preprint in perpetuity. It is made available under a CC-BY 4.0 International license.

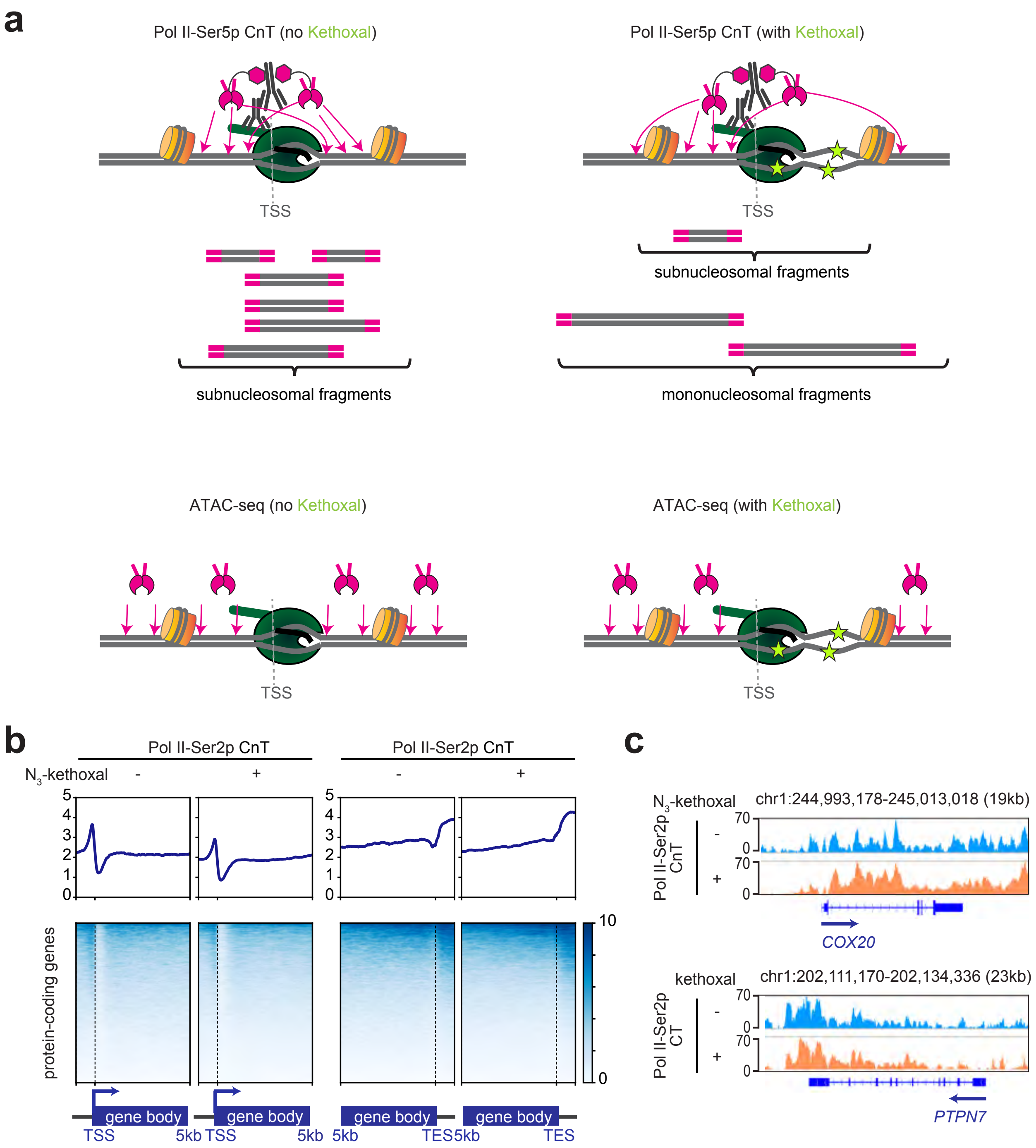

Fig.S3: N3-ethoxal treatment blocks Tn5 integration into ssDNA around TSSs.
a, Schematic illustrating how N_3-kethoxal blocks Tn5 integration into ssDNA between the TSS and the +1 nucleosome, resulting in loss of subnucleosomal fragments while preserving longer fragments from adjacent dsDNA in both CUT&Tag and ATAC-seq. **b,** Heatmaps (bottom) and average plots (top) centered on the TSSs and TESs of 12,397 protein-coding genes for Pol II-Ser2P CnT (±N_3-kethoxal) in K562 cells. Input CUT&Tag library corresponds to N_3-kethoxal-treated Pol II-Ser2P CnT. **c,** Coverage-normalized signals for Pol II-Ser2P CnT (±N_3-kethoxal) in K562 cells.

bioRxiv preprint doi: https://doi.org/10.64898/2026.05.15.725569; this version posted May 19, 2026. The copyright holder for this preprint (which was not certified by peer review) is the author/funder, who has granted bioRxiv a license to display the preprint in perpetuity. It is made available under a CC-BY 4.0 International license.

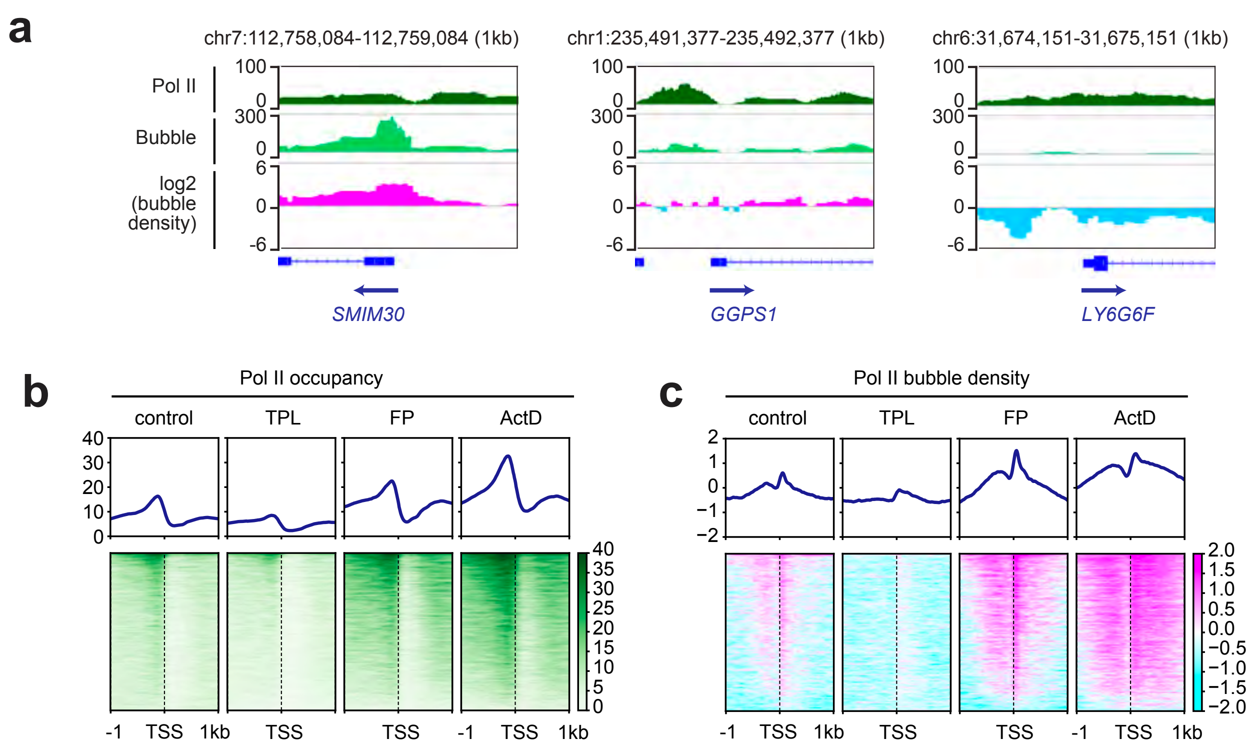

Fig.S4: Promoter-proximal pausing increases bubble density around TSSs.
a, Coverage-normalized signals for Pol II CnT, KAS-CnT, and $\log_2$(Pol II KAS-CnT/CnT) ratios in K562 cells. **b, c,** Heatmaps (bottom) and average plots (top) aligned to the TSSs of 3,859 active genes in K562 under the indicated treatments, showing Pol II occupancy (b) or $\log_2$(Pol II bubble density) ratios (c). Pol II occupancy is quantified by Pol II CnT signals, and bubble density by $\log_2$(Pol II KAS-CnT/CnT) ratios. Each row represents one gene.

bioRxiv preprint doi: https://doi.org/10.64898/2026.05.15.725569; this version posted May 19, 2026. The copyright holder for this preprint (which was not certified by peer review) is the author/funder, who has granted bioRxiv a license to display the preprint in perpetuity. It is made available under a CC-BY 4.0 International license.

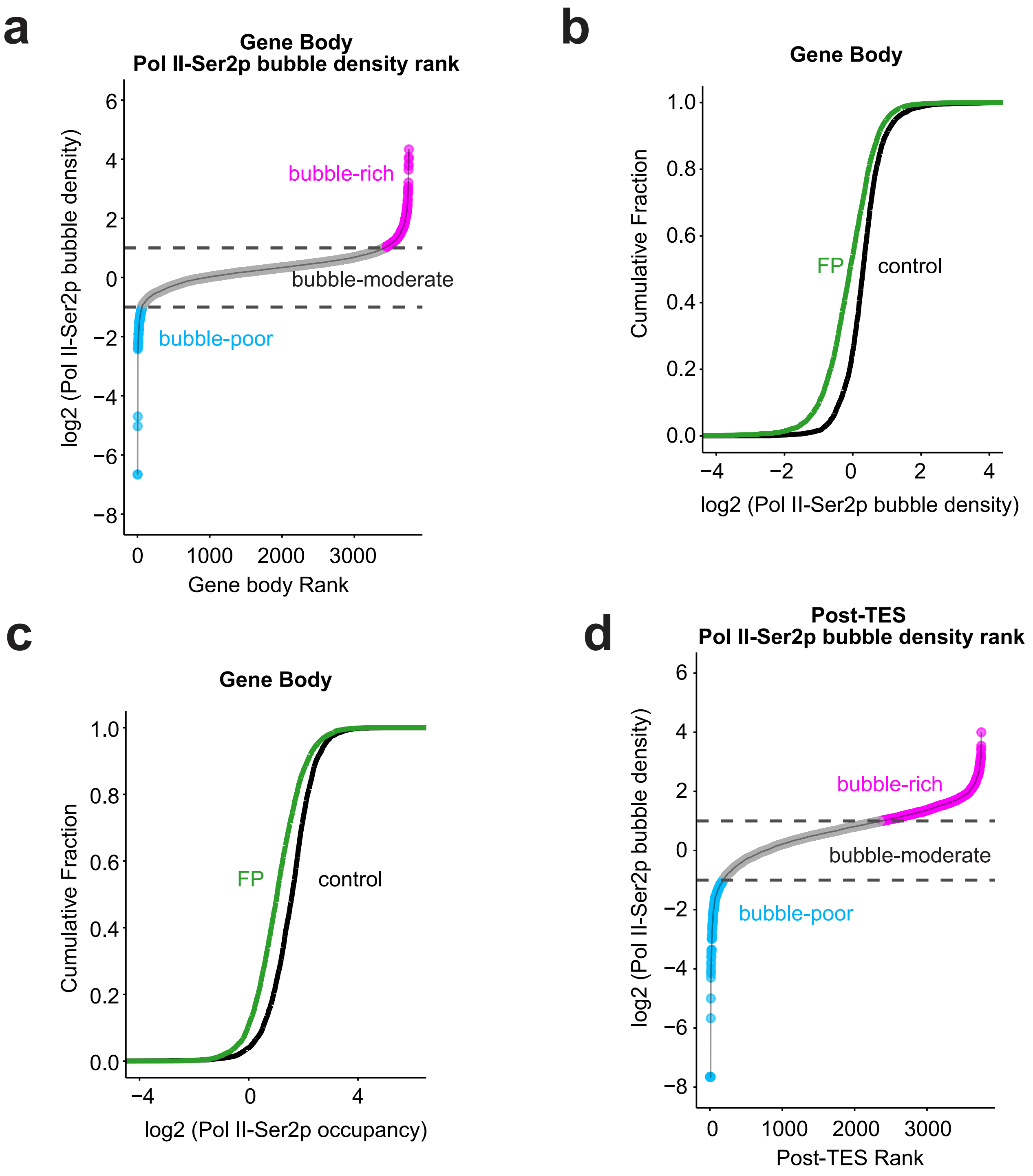

Fig.S5: Bubble density varies across transcribed regions.

a, d, Ranking of 3,754 active genes by transcription bubble density across gene bodies (**a**) or post-TES regions (**d**) in K562 cells, quantified by $\log_2$(Pol II-Ser2P KAS-CnT/CnT) ratios. **b,** Cumulative plots showing Pol II-Ser2P bubble density across gene bodies in K562 under the indicated treatments. **c,** Cumulative plots showing Pol II-Ser2P occupancy across gene bodies in K562 under the indicated treatments.

bioRxiv preprint doi: https://doi.org/10.64898/2026.05.15.725569; this version posted May 19, 2026. The copyright holder for this preprint (which was not certified by peer review) is the author/funder, who has granted bioRxiv a license to display the preprint in perpetuity. It is made available under a CC-BY 4.0 International license.

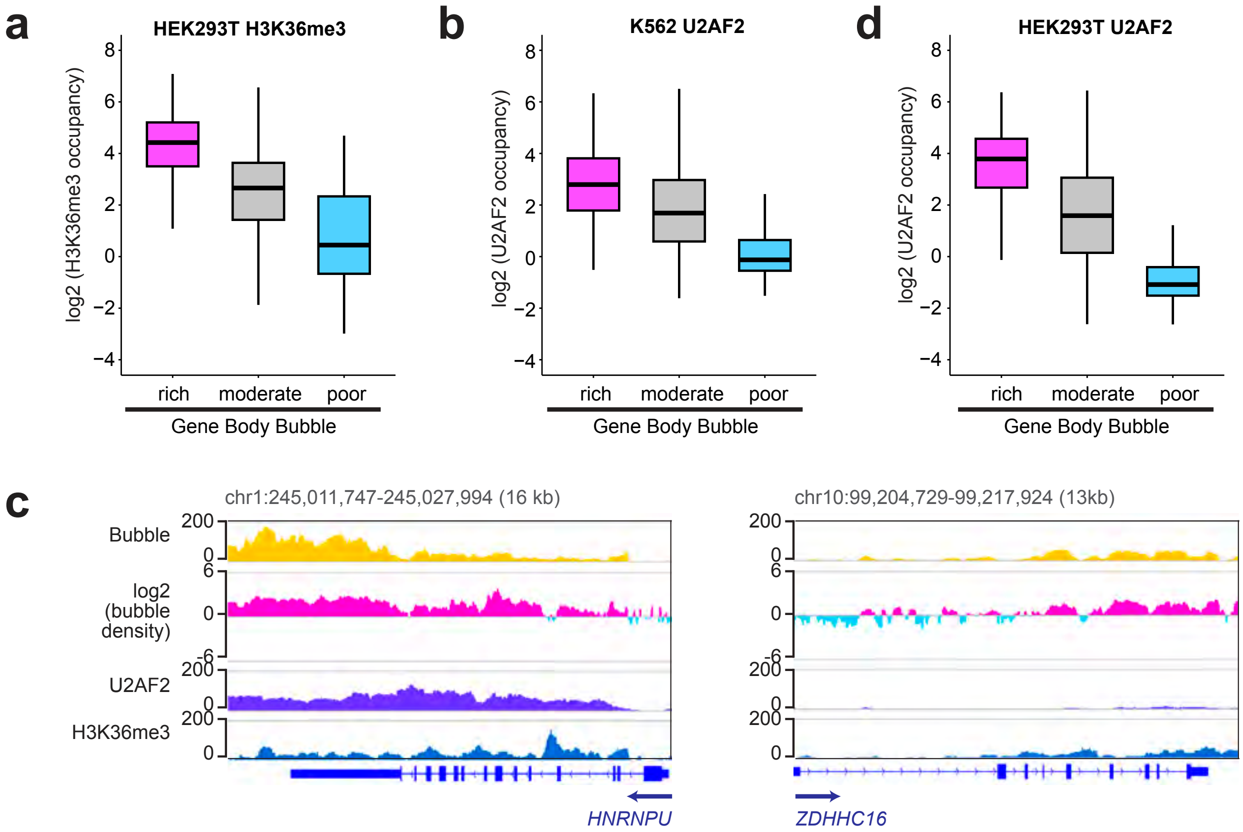

Fig.S6: Elongating Pol II engagement is associated with H3K36me3 and U2AF2 binding. **a,** Coverage-normalized H3K36me3 CnT signals across bubble-rich, -moderate, and -poor gene bodies in HEK293T cells. **b, d,** Coverage-normalized U2AF2 CnR signals across bubble-rich, -moderate, and -poor gene bodies in K562 (**b**) or HEK293T (**d**) cells. **c,** Coverage-normalized signals for Pol II-Ser2P KAS-CnT, log_2(Pol II-Ser2p KAS-CnT/CnT) ratios, U2AF2 CnR and H3K36me3 CnT in HEK293T cells.

bioRxiv preprint doi: https://doi.org/10.64898/2026.05.15.725569; this version posted May 19, 2026. The copyright holder for this preprint (which was not certified by peer review) is the author/funder, who has granted bioRxiv a license to display the preprint in perpetuity. It is made available under a CC-BY 4.0 International license.

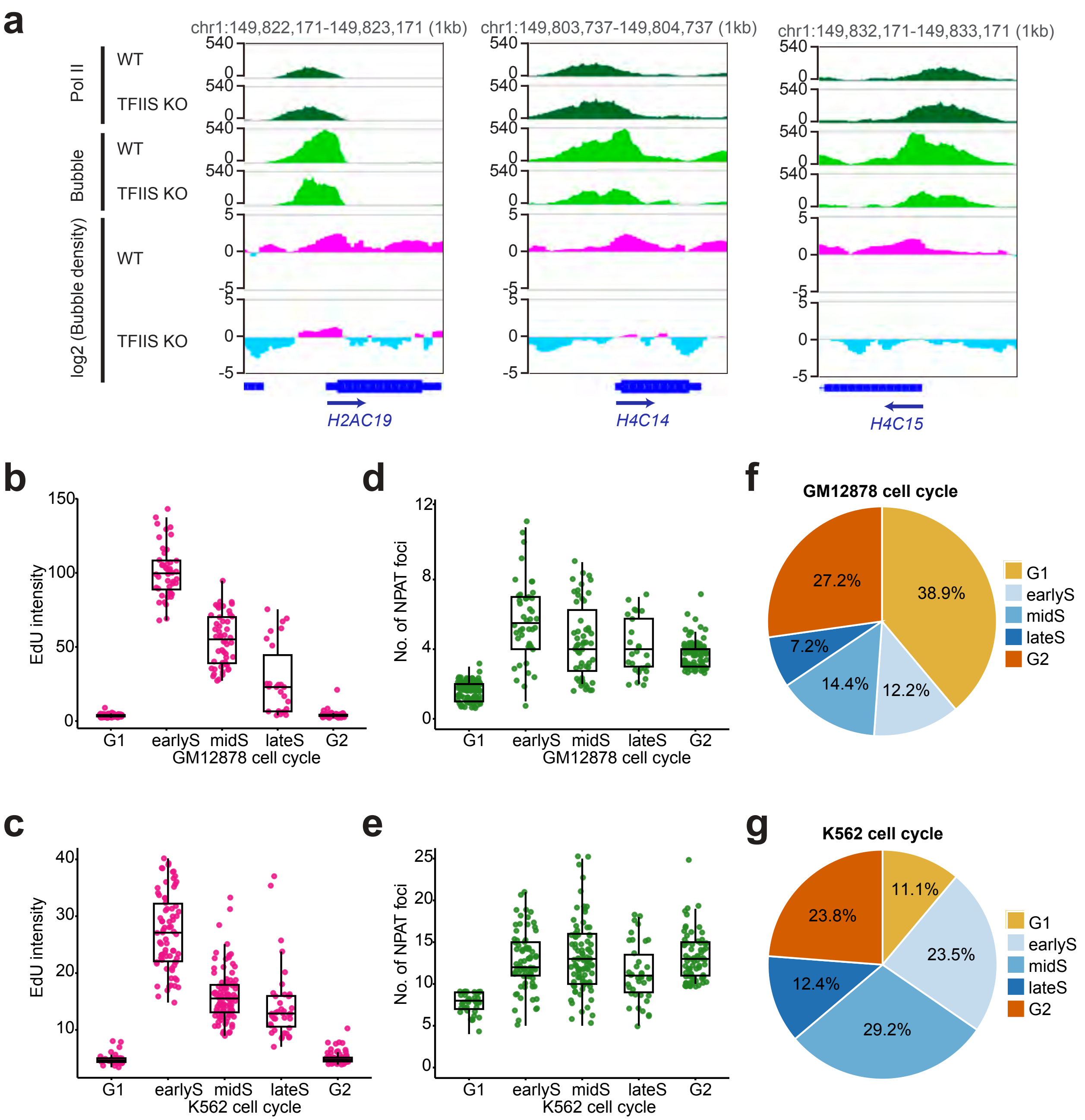

Fig.S7: Dense transcription bubbles persist at RD-histone genes throughout the cell cycle. **a,** Coverage-normalized signals for Pol II CnT, KAS-CnT, and $\log_2$(Pol II KAS-CnT/CnT) ratios in WT and TFIIS KO cells. **b, c,** Mean EdU intensity in GM12878 (**b**) and K562 (**c**) nuclei at the indicated cell-cycle stages. **d, e,** Number of NPAT foci in GM12878 (**d**) and K562 (**e**) nuclei at the indicated cell-cycle stages. **f, g,** Percentage of cells in each cell-cycle stage in GM12878 (**f**) and K562 (**g**). N indicates the number of cells analyzed.

bioRxiv preprint doi: https://doi.org/10.64898/2026.05.15.725569; this version posted May 19, 2026. The copyright holder for this preprint (which was not certified by peer review) is the author/funder, who has granted bioRxiv a license to display the preprint in perpetuity. It is made available under a CC-BY 4.0 International license.

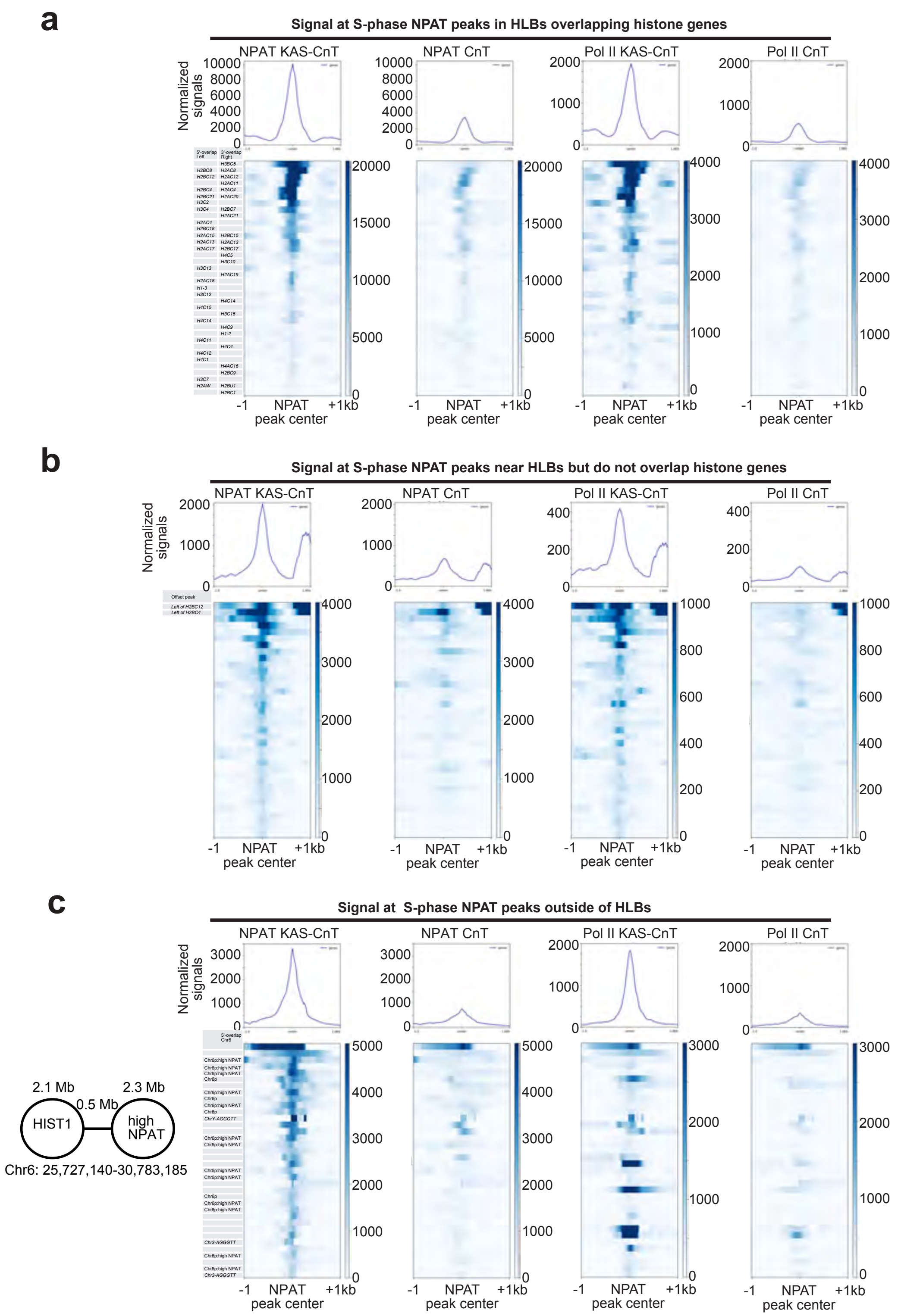

Fig.S8: NPAT is juxtaposed to transcription bubbles within or near HLBs.
a, Heatmaps (bottom) and average plots (top) centered on the 36 NPAT S-phase KAS-CnT peaks overlapping the 5′ ends of RD-histone genes at the HLB, showing Pol II and NPAT KAS-CnT and CnT signals in K562 S-phase cells. **b,** Same as (a), but for the top 36 NPAT peaks (based on NPAT KAS-CnT signal) located within HLBs that do not overlap RD-histone genes. **c,** Same as (a), but for the top 36 NPAT peaks located outside of HLBs.
